# IGF2-tagging of GAA promotes full correction of murine Pompe disease at a clinically relevant dosage of lentiviral gene therapy

**DOI:** 10.1016/j.omtm.2022.09.010

**Published:** 2022-09-24

**Authors:** Qiushi Liang, Fabio Catalano, Eva C. Vlaar, Joon M. Pijnenburg, Merel Stok, Yvette van Helsdingen, Arnold G. Vulto, Ans T. van der Ploeg, Niek P. van Til, W.W.M. Pim Pijnappel

**Affiliations:** 1 Department of Hematology and Research Laboratory of Hematology, West China Hospital, Sichuan University, Chengdu, Sichuan 610041, P. R. China; 2 Department of Clinical Genetics, Erasmus MC University Medical Center, Rotterdam 3015GE, the Netherlands; 3 Department of Pediatrics, Erasmus MC University Medical Center, Rotterdam 3015GE, the Netherlands; 4 Center for Lysosomal and Metabolic Diseases, Erasmus MC University Medical Center, Rotterdam 3015GE, the Netherlands; 5 Department of Hematology, Erasmus MC University Medical Center, Rotterdam 3015GE, the Netherlands; 6 Hospital Pharmacy, Erasmus MC University Medical Center, Rotterdam 3015GE, the Netherlands

**Keywords:** Pompe, glycogen storage disease, gene therapy, lentiviral, hematopoietic, GAA, IGF2

## Abstract

Pompe disease is caused by deficiency of acid α-glucosidase (GAA), resulting in glycogen accumulation in various tissues, including cardiac and skeletal muscles and the central nervous system (CNS). Enzyme replacement therapy (ERT) improves cardiac, motor, and respiratory functions but is limited by poor cellular uptake and its inability to cross the blood-brain barrier. Previously, we showed that hematopoietic stem cell (HSPC)-mediated lentiviral gene therapy (LVGT) with codon-optimized *GAA* (LV-*GAAco*) caused glycogen reduction in heart, skeletal muscles, and partially in the brain at high vector copy number (VCN). Here, we fused insulin-like growth factor 2 (*IGF2*) to a codon-optimized version of *GAA* (LV-*IGF2.GAAco*) to improve cellular uptake by the cation-independent mannose 6-phosphate/IGF2 (CI-M6P/IGF2) receptor. In contrast to LV-*GAAco*, LV-*IGF2.GAAco* was able to completely normalize glycogen levels, pathology, and impaired autophagy at a clinically relevant VCN of 3 in heart and skeletal muscles. LV-*IGF2.GAAco* was particularly effective in treating the CNS, as normalization of glycogen levels and neuroinflammation was achieved at a VCN between 0.5 and 3, doses at which LV-*GAAco* was largely ineffective. These results identify *IGF2.GAA* as a candidate transgene for future clinical development of HSPC-LVGT for Pompe disease.

## Graphical abstract

**Figure:**
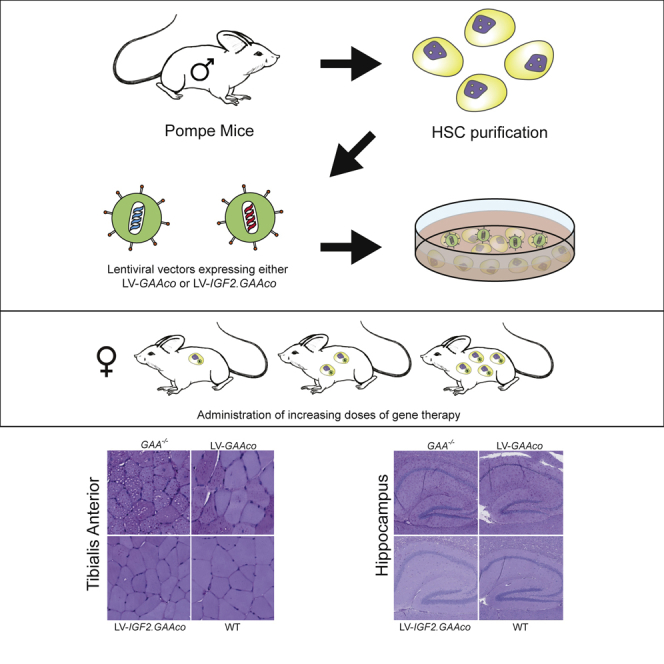

## Teaser

Pompe disease is caused by deficiency of GAA. Lentiviral gene therapy using IGF2-tagged GAA showed superior correction of glycogen levels, pathology, and impaired autophagy in heart, skeletal muscles, and CNS compared with untagged GAA. These results identify *IGF2.GAA* as a candidate for gene therapy for Pompe disease.

## Introduction

Pompe disease, or glycogen storage disease type II (GSDII, OMIM 232300), is an autosomal recessive lysosomal storage disorder (LSD) caused by deficiency of the lysosomal enzyme acid-α glucosidase (GAA). GAA is involved in the catabolism of lysosomal glycogen and its deficiency leads to glycogen accumulation in many tissues, most prominently in cardiac and skeletal muscles.^1^^,^^2^ The most severe form of Pompe disease is the classic infantile form, which manifests shortly after birth with a hypertrophic cardiomyopathy, feeding difficulties, and progressive generalized muscle weakness. If left untreated, these patients die due to cardiorespiratory failure before the age of 1 year.^2^, ^3^, ^4^ Patients with a less severe phenotype can develop symptoms at any age characterized by progressive proximal muscle weakness. Respiratory muscles, and especially the diaphragm, are affected as well. As a consequence, most patients become ventilator dependent and wheelchair-bound at some point of their life.^5^^,^^6^ The heart is rarely involved in these patients.

Enzyme replacement therapy (ERT) with recombinant human GAA (rhGAA), derived from Chinese hamster ovary (CHO) cells, was approved as registered treatment for patients with Pompe disease in 2006 (Myozyme, Genzyme Corporation). Over a decade, ERT has proven to prolong survival, reverse the life-threatening cardiomyopathy, and improve muscle function in patients with Pompe disease. However, ERT also has limitations. It requires a life-long (bi)-weekly administration. A significant proportion of patients die prematurely despite ERT, or show suboptimal clinical response to the therapy.^7^, ^8^, ^9^, ^10^, ^11^, ^12^, ^13^, ^14^, ^15^, ^16^, ^17^, ^18^, ^19^

One reason is poor uptake of intravenously supplied rhGAA by skeletal muscle via the cation-independent mannose 6-phosphate/insulin-like growth factor 2 (IGF2) receptor (CI-M6P/IGF2). As a result, glycogen buildup cannot be completely prevented, and secondary events such as lysosomal pathology, inhibition of autophagy and muscle damage can occur.^8^^,^^20^, ^21^, ^22^ Furthermore, high sustained antibody titers against rhGAA may interfere with the efficacy of ERT by inhibiting cellular uptake and/or enzymatic activity.^23^^,^^24^ Moreover, ERT cannot pass the blood-brain barrier (BBB) and is therefore unable to overcome the cognitive problems that have been described as a new emerging feature of classic infantile Pompe patients.^8^^,^^25^, ^26^, ^27^, ^28^, ^29^, ^30^, ^31^

In order to address the limitations of ERT, new treatments such as gene therapy are under development. Gene therapy is in the spotlight since it offers the possibility to correct Pompe disease at its genetic roots, restoring an endogenous and long-lasting production of GAA.^32^ Several ongoing gene therapy clinical trials for Pompe disease are based on adeno-associated vectors (AAV) with tropisms for liver or muscles (NCT03533673, NCT02240407, NCT00976352, NCT04174105, NCT04093349).^33^, ^34^, ^35^ These approaches represent promising new treatment options for Pompe disease, although there are some limitations, such as the pre-existing adaptative immunity to the AAV capsid proteins and the limited efficacy in the brain.^36^, ^37^, ^38^, ^39^ Moreover, AAV transduction of actively replicating cells leads to vector dilution over time, posing a problem for the treatment of classic infantile Pompe patients.^39^

Hematopoietic stem cell (HSPC)-mediated lentiviral gene therapy (LVGT) may serve as an alternative therapeutic option to treat Pompe disease. This therapy involves transplantation of *ex vivo* gene-modified autologous HSPCs aimed to overexpress the therapeutic transgene. The principle is based on secretion of the enzyme into the circulation, followed by binding to the CI-M6P/IGF2 and transport to the lysosome in affected tissues.^40^^,^^41^ So far, HSPC-LVGT has demonstrated long-lasting clinical benefits in several clinical trials for different inherited disorders.^42^, ^43^, ^44^, ^45^ In addition, clinical trials for metachromatic leukodystrophy (MLD)^46^^,^^47^ and X-linked adrenoleukodystrophy (X-ALD),^45^^,^^48^ two metabolic disorders with a prominent CNS involvement, have shown the potential of *ex vivo* lentiviral gene therapy to treat the CNS.

We and others have previously demonstrated that HSPC-LVGT ensured long-term engraftment, providing a continuous supply of GAA enzyme after a single intervention in *Gaa*^*−/−*^ mice, leading to increased levels of GAA enzyme activity in affected tissues and to improved cardiac and motor function. However, phenotypic correction was only observed at high vector copy number (VCN), and did not effectively reduce glycogen to normal levels in heart, skeletal muscles,^49^^,^^50^ or the brain.^49^^,^^50^ We recently reported that codon optimization of GAA improved the efficacy of gene therapy; however, correction was achieved at a high VCN and, especially, there was no full clearance of glycogen in the brain.^51^ The probability of genotoxicity events after transduction with third-generation lentiviral vectors increases with the number of integration events. In common clinical practice, a VCN below 4 is considered to be safe.^42^^,^^45^, ^46^, ^47^^,^^52^, ^53^, ^54^, ^55^, ^56^ This makes our previous effort to treat murine Pompe disease with LV-*GAAco* lentiviral gene therapy likely unsuitable for clinical application.^51^

For these reasons, we sought to increase the therapeutic efficacy and safety of HSPC-LVGT by enhancing uptake of GAA into affected tissues. M6P moieties on lysosomal proteins are able to bind to the CI-M6P/IGF2. Alternatively, IGF2 can dock to CI-M6P/IGF2 through a different binding site, but with a much higher affinity than M6P.^57^ This has previously resulted in the design of an IGF2-tagged rhGAA chimeric protein that, compared with untagged rhGAA, showed superior clearance of glycogen after intravenous injection in a Pompe disease mouse model.^58^ These results led us to modify our previously described lentiviral vector to contain a codon-optimized *GAA* sequence fused to codon-optimized human *IGF2* (LV-*IGF2.GAAco*). A dose-response analysis revealed that LV-*IGF2.GAAco* corrects glycogen accumulation, pathology, expression of autophagy markers, and motor function at a much lower VCN compared with LV-*GAAco* in all the tissues analyzed. In addition, HSPC-LVGT with LV-*IGF2.GAAco* resulted in complete normalization of brain glycogen content, neuroinflammation, and CNS pathology.

## Results

### Efficient uptake of IGF2.GAA by primary *Gaa*^−/−^ murine myotubes

**Figure 1:**
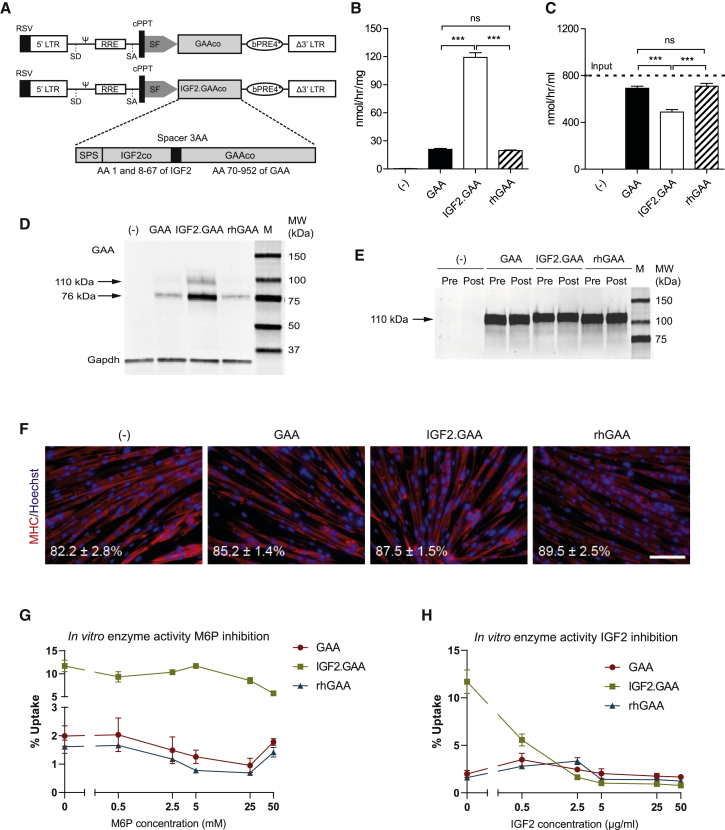
*In vitro* characterization of GAA and IGF2.GAA proteins used in HSPC-LVGT (A) pRRL third-generation lentiviral vectors encoding either codon-optimized human GAA (GAAco) or IGF2-tagged GAA (the components of *IGF2.GAAco* are shown; SPS, signal peptide sequence of IGF2; AA, amino acid) under the spleen focus-forming virus (SFFV) promoter. (B–F) Enzyme activity in cells (B) and media (C) after uptake of IGF2.GAA, GAA, or rhGAA in *Gaa*^−/−^ mouse-derived myotubes. Dashed line in (B) represents the input concentration used for the experiments in (B) and (C). (D and E) Immunoblot analysis of the experiment shown in (B) and (C) using an antibody to GAA. (D) Media before (Pre) and after (Post) uptake. (F) *Gaa*^−/−^ mouse-derived myotubes after uptake stained for myosin heavy chain (MHC) in red and nuclei (using Hoechst) in blue. Fusion index is indicated at the bottom left corner. Representative images are shown. Scale bar, 100 μm. (G and H) GAA, IGF2.GAA, or rhGAA uptake in *Gaa*^−/−^ mouse-derived myotubes in the presence of increasing concentrations of mannose-6-phosphate (M6P) (G) or IGF2 (H). Data are expressed as percentage of activity in the input medium. Data represent means ± SEM and are analyzed by one-way ANOVA followed by Bonferroni’s multiple testing correction. n = 3 biological replicates/condition. ∗∗∗p ≤ 0.001; ns, not significant. Comparisons are indicated by brackets.

We generated third-generation self-inactivating (SIN) lentiviral vectors encoding codon-optimized human *GAA* (LV-*GAAco*) and a chimeric protein containing the IGF2 signal peptide sequence, amino acids (AA) 1 fused to AA 8–67 of codon-optimized mature human IGF2, a Gly-Ala-Pro spacer sequence, and AA 70–952 of codon-optimized human GAA (LV-*IGF2.GAAco*). The *IGF2.GAAco* translates into the same amino acid sequence as previously described for glycosylation-independent lysosomal targeting (GILT) ERT (Figure 1A).^58^ To assess the effect of the IGF2 tag on GAA enzyme activity, HEK 293T cells were transduced with either LV-*GAAco* or LV-*IGF2.GAAco*. After correction for VCN, GAA activity per integrated copy was comparable between LV*-GAAco* and LV*-IGF2.GAAco* transduced cells, indicating that the addition of the IGF2 tag did not interfere with GAA enzyme activity (472.8 ± 27.1 versus 388.2 ± 4.0 nmol/h/mg/VCN; Figure S1), as previously reported.^58^

Secretion of GAA and IGF2.GAA proteins are driven by the human GAA signal peptide and by the human IGF2 signal peptide, respectively. To evaluate differences in secretion, we transiently transfected HEK 293T cells with pcDNA3.1 expressing either GAA or IGF2.GAA and measured secretion over 4 days after transfection (Figure S2). At day 4 after transfection, intracellular and secreted GAA protein levels were higher in cells expressing human GAA than in cells expressing IGF2.GAA (Figure S2B). This difference could not be explained by a difference in transfection efficiency, suggesting a lower protein expression from the pcDNA3.1 IGF2.GAA construct (Figure S2D). The percentage of secreted enzyme activity (Figure S2A) was comparable for GAA and IGF2.GAA over the course of 4 days. The activity levels in medium at day 4 after transfection correlated with protein levels for both GAA and IGF2.GAA (Figure S2C). This shows that GAA or IGF2 signal peptides have the same efficacy in driving secretion of the respective proteins.

Next, we studied the uptake of IGF2.GAA protein in primary *Gaa*^−/−^ murine myotubes *in vitro*. Conditioned medium from LV*-GAAco* or LV*-IGF2.GAAco*-transduced HEK 293T cells was applied to myotubes at an initial input concentration of 800 nmol/h/mL GAA activity (Figure 1B, dashed line). rhGAA (Myozyme) was administered at the same input concentration and used as a control. Following 24 h of incubation, 120 nmol/h/mg IGF2.GAA, corresponding to 15% of input, was taken up by the cells, compared with 20 nmol/h/mg (2.5% of input) of GAA or rhGAA (Figure 1B). This was also reflected by the residual amount of GAA activity in the medium following incubation: IGF2.GAA containing medium showed significantly larger reduction of GAA activity compared with GAA and rhGAA-containing media (Figure 1C). In agreement, immunoblot analysis in cell lysates showed higher GAA protein levels in IGF2.GAA-treated cells compared with cells treated with GAA and rhGAA. After uptake, GAA was predominantly present as the 76-kDa active form, indicating that adequate intracellular processing and transport to the lysosomes had occurred in all the treatment conditions. In the media, only the 110-kDa precursor was detectable before and after the treatment (Figures 1D and 1E). We note that the precursor of IGF2.GAA was slightly larger due to the presence of the N-terminal IGF2 epitope tag (Figure 1D).

The quality of myotubes after treatment was assessed by immunofluorescent analysis of myosin heavy chain (MHC), as well as by measurement of the fusion index (Figure 1F). Myotube morphology, immunoreactivity with anti-MHC antibody, and fusion index (∼80%) were comparable across the different conditions, suggesting that myotube differentiation was not influenced by any of the GAA preparations. We next investigated the specificity of IGF2.GAA for the IGF2-binding domain (domain 11) of the CI-M6P/IGF2 receptor.^58^ To this end, uptake of IGF2.GAA was tested in primary *Gaa*^−/−^ murine myotubes as described above, but now in the presence of increasing concentrations of M6P (Figure 1G) or recombinant human IGF2 (Figure 1H). M6P inhibited uptake of GAA and rhGAA in a dose-dependent manner starting at 2.5 mM M6P. In contrast, M6P failed to inhibit uptake of IGF2.GAA. Inhibition with IGF2 resulted in a dose-dependent reduction of uptake of IGF2.GAA of 52% at 0.07 μM and 90% at 7 μM, whereas effects on uptake of GAA and rhGAA were negligible (Figure 1H). These results confirm that the IGF2.GAA protein expressed by LV*-IGF2.GAAco* mediates uptake through its cognate binding site on the CI-M6P/IGF2 receptor on mouse skeletal muscle cells, resulting in superior cellular uptake compared with both GAA and rhGAA.

### Full correction of skeletal muscles with LV*-IGF2.GAAco* lentiviral gene therapy at low VCN

**Figure 2:**
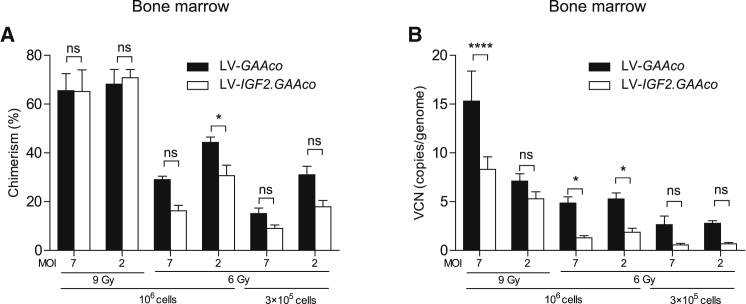
VCN and chimerism in bone marrow after gene therapy (A) Chimerism, expressed as the percentage of reconstituted male donor cells in bone marrow of female recipients treated with gene therapy, determined by qPCR on *Sry* and *Gapdh* loci. (B) VCN measured in bone marrow by qPCR on *HIV* and *Gapdh* loci. VCN is not normalized for chimerism. MOI, multiplicity of infection; Gy, gray. Data represent means ± SEM and are analyzed by two-way ANOVA followed by Bonferroni’s multiple testing correction, using vector (LV*-GAAco* or LV-*IGF2.GAAco*) and gene therapy dose as categorical variables. Significant results are indicated by brackets. Results of multiple comparison analysis are reported in Table S3. LV-*GAAco* and LV-*IGF2.GAAco*, n = 7 per group; KO, n = 5; WT, n = 5. ns, not significant; ∗p ≤ 0.05, ∗∗∗∗p ≤ 0.0001.

We compared LV*-IGF2.GAAco* with LV*-GAAco* for their ability to correct glycogen accumulation, autophagic buildup, and muscle function in skeletal muscles. To this end, we transplanted lentiviral transduced HSPCs into 2-month-old irradiated *Gaa*^−/−^ mice and performed a dose-response analysis by varying lentiviral vector dose, transplanted cell number, and irradiation dose (conditions are presented in Table 1). Analysis was performed 6 months after transplantation. Analysis of bone marrow from both LV*-GAAco* and LV-*IGF2.GAAco*-treated mice showed reconstitution of transplanted gene-modified cells, with VCN and chimerism levels varying according to the gene therapy dose administrated (Figures 2A and 2B). A higher chimerism and VCN for LV-*GAAco* compared with LV*-IGF2.GAAco* was observed at 6 Gy, but no significant difference was observed between those groups when irradiated at 9 Gy.

**Table 1:** Layout of different treatment groups

Group	Treatment	MOI	Transplanted cells	Irradiation (Gy)	N
1	LV-*GAAco*/LV-*IGF2.GAAco*	7	10^6^	9	10
2	LV-*GAAco*/LV-*IGF2.GAAco*	2	10^6^	9	10
3	LV-*GAAco*/LV-*IGF2.GAAco*	7	10^6^	6	10
4	LV-*GAAco*/LV-*IGF2.GAAco*	2	10^6^	6	10
5	LV-*GAAco*/LV-*IGF2.GAAco*	7	3 × 10^5^	6	10
6	LV-*GAAco*/LV-*IGF2.GAAco*	2	3 × 10^5^	6	10
7	Untreated GAA^−/−^	7	NA	NA	6
8	Untreated WT	2	NA	NA	6

MOI, multiplicity of infection; Gy, gray; *Gaa*^−/−^ knockout; WT, FVB/N wild type; NA, not applicable.

GAA enzyme activities in tissue lysates showed dose-dependent increases in all tissues examined (Table 2). In general, GAA activities in tissue lysates from LV-*GAAco*-treated mice were several-fold higher compared with LV-*IGF2.GAAco*-treated mice. Activity levels correlated with GAA protein levels, as determined by immunoblot analyses using a human GAA antibody, in tissue lysates of the diaphragm, quadriceps femoris, gastrocnemius, heart, and tibialis anterior from mice treated with a high dose of gene therapy (Figures S4A–S4I).The molecular weight of the active GAA protein (76 kDa) appeared slightly higher after treatment with LV-*GAAco* than after treatment with LV-*IGF2.GAAco* as determined by SDS-PAGE electrophoresis. We confirmed this by repeating the immunoblot analysis in which we loaded 10-times lower amounts of total protein for LV-*GAAco* compared with LV-*IGF2.GAAco-*treated tissues, and by running the gel for a longer period to increase the resolution (Figures S4J–S4L). We speculate that this may reflect differential intracellular processing and/or post-translational modification, which should be confirmed in future work.

**Table 2:** Tissue GAA activity 6 months after gene therapy

Tissue	LV-GAAco MOI 7–9 Gy 10^6^ cells	LV-IGF2.GAAco MOI 7–9 Gy 10^6^ cells	LV-GAAco MOI 2–9 Gy 10^6^ cells	LV-IGF2.GAAco MOI 2–9 Gy 10^6^ cells	LV-GAAco MOI 7–6 Gy 10^6^ cells	LV-IGF2.GAAco MOI 7–6 Gy 10^6^ cells	LV-GAAco MOI 2–6 Gy 10^6^ cells	LV-IGF2.GAAco MOI 2–6 Gy 10^6^ cells	LV-GAAco MOI 7–6 Gy 3 × 10^5^ cells	LV-IGF2.GAAco MOI 7–6 Gy 3 × 10^5^ cells	LV-GAAco MOI 2–6 Gy 3 × 10^5^ cells	LV-IGF2.GAAco MOI 2–6 Gy 3 × 10^5^ cells	KO	WT
Bone marrow	1,059.01 ± 437.97	116.40 ± 28.40	390.08 ± 52.86	93.31 ± 11.05	374.23 ± 166.00	24.39 ± 4.14	339.49 ± 83.63	37.38 ± 7.58	247.49 ± 172.07	6.75 ± 2.14	124.45 ± 36.80	16.20 ± 3.83	0.78 ± 0.05	4.49 ± 0.37
Leukocytes	1,085.07 ± 481.45	67.11 ± 13.14	329.69 ± 11.44	55.73 ± 7.68	378.84 ± 183.18	8.44 ± 1.37	307.45 ± 35.56	16.74 ± 2.03	76.35 ± 16.78	2.89 ± 0.64	144.71 ± 23.13	5.39 ± 0.72	0.45 ± 0.03	1.84 ± 0.214
Heart	104.48 ± 30.55	9.96 ± 0.49	32.20 ± 1.42	6.31 ± 0.40	39.93 ± 7.66	2.74 ± 0.15	33.45 ± 2.64	3.72 ± 0.29	21.02 ± 2.90	1.68 ± 0.13	18.22 ± 1.80	2.16 ± 0.09	0.94 ± 0.03	15.75 ± 1.201
Tibialis anterior	41.72 ± 2.33	7.25 ± 0.27	20.04 ± 1.06	5.91 ± 0.47	21.57 ± 3.32	3.76 ± 0.16	19.11 ± 1.13	3.63 ± 0.20	11.53 ± 1.32	2.87 ± 0.21	12.82 ± 0.89	3.14 ± 0.12	1.95 ± 0.07	5.20 ± 0.195
Quadriceps femoris	78.96 ± 10.55	5.68 ± 0.20	41.12 ± 3.40	4.65 ± 0.38	30.37 ± 5.76	3.34 ± 0.08	31.91 ± 2.47	3.59 ± 0.28	16.80 ± 1.89	2.68 ± 0.17	18.87 ± 2.40	3.38 ± 0.22	2.17 ± 0.07	6.40 ± 0.251
Gastrocnemius	53.11 ± 6.00	4.97 ± 0.21	21.07 ± 1.84	4.58 ± 0.26	20.09 ± 4.47	2.46 ± 0.07	15.69 ± 1.68	2.62 ± 0.15	10.25 ± 0.88	2.06 ± 0.06	11.90 ± 1.12	2.45 ± 0.04	1.93 ± 0.04	6.36 ± 0.224
Diaphragm	122.93 ± 18.69	9.61 ± 1.03	48.91 ± 4.34	7.43 ± 0.64	42.48 ± 9.98	4.03 ± 0.12	31.97 ± 3.05	4.21 ± 0.26	18.53 ± 2.08	3.11 ± 0.18	23.66 ± 2.15	3.78 ± 0.12	2.29 ± 0.09	9.19 ± 0.322
Cerebrum	5.11 ± 0.21	2.02 ± 0.06	3.47 ± 0.42	1.85 ± 0.05	2.25 ± 0.10	1.85 ± 0.02	2.49 ± 0.08	1.90 ± 0.07	2.18 ± 0.06	1.80 ± 0.04	2.16 ± 0.11	1.83 ± 0.03	1.61 ± 0.03	18.74 ± 0.942
Cerebellum	7.62 ± 0.52	2.55 ± 0.08	4.62 ± 0.37	2.44 ± 0.14	3.37 ± 0.20	1.96 ± 0.05	3.53 ± 0.20	1.99 ± 0.07	2.78 ± 0.09	2.02 ± 0.06	2.99 ± 0.21	1.93 ± 0.09	2.00 ± 0.04	15.46 ± 0.735

Activity is expressed in nmol/h/mg of total protein.

Depending on the irradiation dose, the number of transplanted cells, and the multiplicity of infection (MOI), GAA activities exceeded those of knockout mice in bone marrow, leukocytes, and cardiac and skeletal muscles. In lysates from cerebrum and cerebellum, increases in GAA activity were modest (LV-*GAAco*) to low (LV-*IGF2.GAAco*). There was a lack of correlation between GAA activities in tissue lysates and glycogen content (see below).

**Figure 3:**
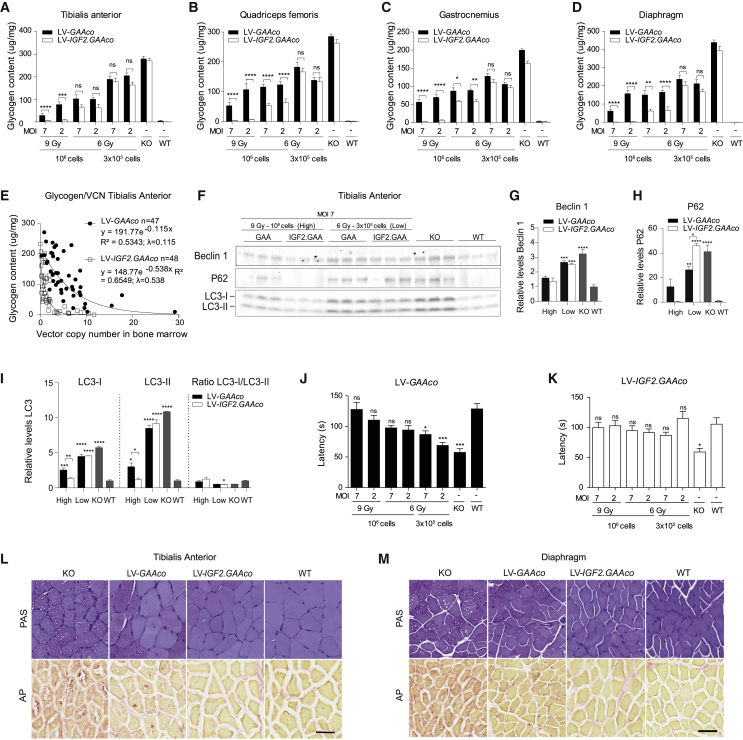
Gene therapy with LV-*IGF2.GAAco* results in correction in skeletal muscles (A–D) Total glycogen content in skeletal muscles after different doses of gene therapy with LV-*IGF2.GAAco* or LV-*GAAco*. MOI, multiplicity of infection; Gy, gray. (E) Correlation between bone marrow VCN and glycogen clearance in tibialis anterior. λ, exponential decay constant. VCN is not normalized for chimerism. (F–I) Immunoblot analysis in biological triplicates of tibialis anterior of mice treated with a high (MOI 7, 9 Gy, 10^6^ cells) or low dose (MOI 7, 6 Gy, 3 × 10^5^ cells) of gene therapy using antibodies against beclin 1, p62, and LC3. Density levels of beclin 1 (G), p62 (H), or LC3 (I) are quantified from (F) (loading controls are shown in Figure S5A). In (G)–(I), values are relative to WT. (J and K) Latency on a rotarod after gene therapy with LV-*GAAco* or (J) LV-*IGF2.GAAco* (K). (L and M) Representative images of PAS (upper row) and AP (lower row) stainings in tibialis anterior (L) and diaphragm (M) after high-dose gene therapy. Scale bar, 50 μm. Data are presented as means ± SEM. In (A)–(D), data are analyzed by two-way ANOVA with Bonferroni’s correction, using vector (LV*-GAAco* or LV-*IGF2.GAAco*) and gene therapy dose as categorical variables. Significant results are indicated by brackets. Results of multiple comparison analysis are reported in Table S3. In (G)–(K), data are analyzed by one-way ANOVA followed by Bonferroni’s multiple testing. Significance is expressed as relative to WT; other significant comparisons are indicated by brackets. (A–D; J, K) LV*-GAAco* and LV-*IGF2.GAAco*, n = 7; KO, n = 5; WT, n = 5. (G–I) n = 3 for all groups. (L and M) LV*-GAAco* and LV-*IGF2.GAAco*, n = 3 per group; KO, n = 2; WT, n = 2. ns, not significant; ∗p ≤ 0.05, ∗∗p ≤ 0.01, ∗∗∗p ≤ 0 .001, ∗∗∗∗p ≤ 0 .0001.

Next, we evaluated glycogen content after gene therapy. Age-matched untreated *Gaa*^−/−^ mice showed pronounced glycogen accumulation in skeletal muscles compared to wild-type (WT) control animals (Figures 3A–3D). After gene therapy, all treatment groups showed a significant dose-dependent reduction of glycogen content in the tibialis anterior, quadriceps femoris, gastrocnemius, and diaphragm, but only gene therapy with LV*-IGF2.GAAco* resulted in full correction of glycogen content to WT levels (Figures 3A–3D, p ≤ 0.001). Glycogen content in skeletal muscles versus VCN in bone marrow was plotted for all mice in the experiment and described by an exponential decay function, in which the decay constant (λ) relates to the reduction of glycogen levels per vector copy. Mice treated with LV*-IGF2.GAAco* gene therapy showed a λ of 0.538, while LV*-GAAco* treatment led to a λ of 0.115, indicating that glycogen content per VCN was significantly more reduced in LV*-IGF2.GAAco* than in LV*-GAAco*-treated animals (Figure 3E; for statistical outcome see Table S2). Correction of glycogen content was achieved at a VCN in bone marrow as low as 3 after lentiviral gene therapy with LV*-IGF2.GAAco*. In a direct comparison at subtherapeutic conditions, the clinically acceptable MND promoter^59^, ^60^, ^61^ showed similar efficacy in reducing glycogen levels compared with the SF promoter (Figure S10).

We next analyzed autophagy markers after gene therapy. Tibialis anterior homogenates from high-dose (MOI 7, 9 Gy, 10^6^ transplanted cells) and low-dose (MOI 7, 6 Gy, 3 × 10^5^ transplanted cells) gene-therapy-treated mice were assessed by immunoblot analysis using antibodies to beclin 1, microtubule-associated protein 1 light chain 3 alpha (LC3), and p62 (SQSTM1/p62) (Figure 3F; see Figure S5 for loading controls). *Gaa*^−/−^ mice presented a ∼5- and ∼10-fold increase of LC3-I and LC3-II levels, respectively (Figure 3I). Effects on the LC3-I/LC3-II ratio were small to not significant. In addition, beclin 1 and p62 levels were elevated three and 40 times in *Gaa*^−/−^ compared with WT mice, respectively (Figures 3G and 3H). These results are consistent with impaired autophagy, as previously reported in *Gaa*^−/−^ mice.^62^ Gene therapy treatment at low dose with either LV-*GAAco* or LV-*IGF2.GAAco* had low to no effect on expression of the autophagic markers analyzed despite the small reduction of glycogen levels at these treatment conditions (Figures 3F–3I versus 3A). In contrast, full normalization of expression of all autophagic markers tested was achieved with high-dose gene therapy using LV*-IGF2.GAAco*, while LV*-GAAco* only partially normalized expression of autophagic markers (Figures 3F–3I). These results parallel glycogen content measured after gene therapy in these treatment conditions, in which LV-*IGF2.GAAco* but not LV*-GAAco* treatment fully normalized glycogen content (Figure 3A).

To assess motor function, we performed rotarod measurements 6 months after transplantation (Figures 3J and 3K). Mice treated with LV*-GAAco* or LV-*IGF2.GAAco* were tested in two separate sessions with *Gaa*^−/−^ and WT controls. After gene therapy at high or moderate dose (9 or 6 Gy, 10^6^ transplanted cells), we observed no differences in the latency to fall between WT and treated animals in both LV-*IGF2.GAAco* and LV-*GAAco* cohorts (Figures 3J and 3K). Of note, animals treated with LV-*IGF2.GAAco* gene therapy at MOI 2, 6 Gy, and 3 × 10^5^ transplanted cells showed higher latency to fall compared with the corresponding LV-*GAAco* group, which may be explained by the significantly different average body weight of some mice within this group (LV-*IGF2.GAAco*, 17.0 ± 0.5 g versus LV-*GAAco*, 23.6 ± 0.4 g; no difference in the average body weight was detected between the other gene therapy groups; Figure S11).^63^

Periodic acid-Schiff (PAS) and acid phosphatase (AP) stainings were performed on sections of the tibialis anterior (Figure 3L) and diaphragm (Figure 3M) to assess muscle pathology and glycogen content after high-dose gene therapy (MOI 7, 9 Gy, 10^6^ transplanted cells). Muscle of *Gaa*^−/−^ mice showed vacuolization and PAS-positive fibers (Figures 3L and 3M upper row). In line with this, muscle from *Gaa*^−/−^ mice stained positive for acid phosphatase, a sensitive marker used in patients with Pompe disease to visualize enlarged lysosomes (Figures 3L and 3M lower row).^64^ Gene therapy with LV-*IGF2.GAAco* fully normalized vacuolization and reactivity for PAS and AP in tibialis anterior and diaphragm, whereas treatment with LV*-GAAco* led to diminished vacuolization, glycogen content, and AP-positive areas only in a subset of muscle fibers (Figures 3L, 3M, S6, and S7).

Taken together, these results demonstrate superior efficacy of IGF2-tagged GAA over GAA when used as lentiviral gene therapy to treat skeletal muscle pathology in a Pompe disease mouse model. In contrast to gene therapy using *GAAco*, full normalization of glycogen content and expression of autophagic markers was achieved in LV*-IGF2.GAAco*-treated animals.

### Full correction of the heart with LV-*IGF2.GAAco* lentiviral gene therapy at low VCN

**Figure 4:**
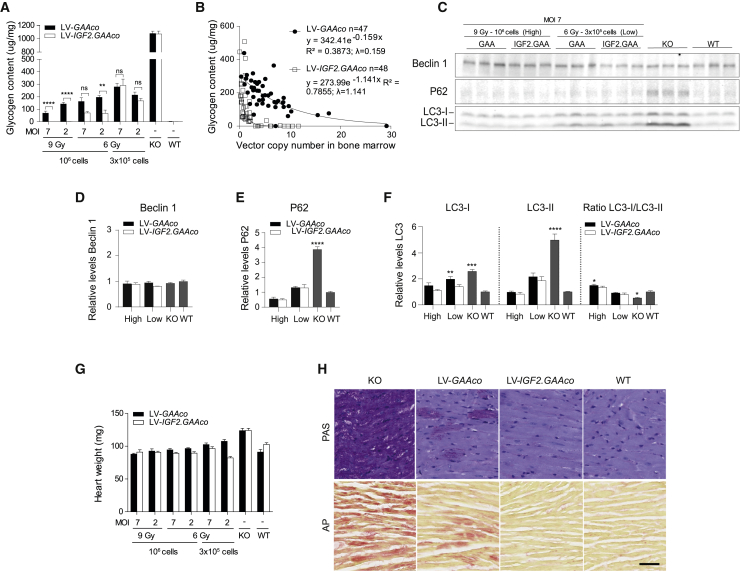
Gene therapy results in correction in heart (A) Total glycogen content in cardiac muscle after different doses of gene therapy. MOI, multiplicity of infection; Gy, gray. (B) VCN in bone marrow versus glycogen content after gene therapy. λ, exponential decay constant. VCN is not normalized for chimerism. (C–F) Immunoblot analysis of heart lysate with antibody to beclin 1, p62, and LC3 after high-dose or low-dose gene therapy (C). Density levels of beclin 1 (D), p62 (E), and LC3 (F) were quantified from C (total protein control is shown in Figure S3B). In (D)–(F), quantification density values are relative to WT value (set as 1). (G) Heart wet weight after gene therapy. (H) PAS (upper row) and AP (lower row) stainings in cardiac tissue from mice treated with high-dose gene therapy. Scale bar, 50 μm. Data are presented as means ± SEM. In (A) and (G), data are analyzed by two-way ANOVA with Bonferroni’s correction, using vector (LV*-GAAco* or LV-*IGF2.GAAco*) and gene therapy dose as categorical variables. Significant results are indicated by brackets. Results of multiple comparison analysis are reported in Table S3. Western blot quantifications (D–F) are analyzed by one-way ANOVA followed by Bonferroni’s multiple testing. Significance is expressed as relative to WT levels; other significant comparisons are indicated by brackets. (A and G) LV-*GAAco* and LV-*IGF2.GAAco*, n = 7; KO, n = 5; and WT, n = 5. (D-F) n = 3. In (H), LV*-GAAco* and LV-*IGF2.GAAco*, *n* = 3 per group; KO, n = 2; and WT, n = 2. ns, not significant; ∗p ≤ 0.05, ∗∗p ≤ 0.01, ∗∗∗p ≤ 0 .001, ∗∗∗∗p ≤ 0 .0001.

Eight-month-old *Gaa*^−/−^ mice showed pronounced glycogen accumulation in the heart (Figure 4A). As in skeletal muscle, we observed a dose-dependent reduction of the glycogen content in heart after gene therapy, with a more pronounced effect in mice treated with LV-*IGF2.GAAc*o gene therapy. In particular, at low dose (6 Gy, 3 × 10^5^ transplanted cells), gene therapy with LV-*IGF2.GAAco* and LV-*GAAco* were equally effective, while at a higher dose (6 Gy, 10^6^ transplanted cells) LV-*IGF2.GAAco* was two to three times more efficient compared with LV-*GAAco*. At the highest gene therapy dose (9 Gy, 10^6^ transplanted cells), LV-*IGF2.GAAco* fully normalized glycogen to WT levels, whereas LV-*GAAco* caused a partial correction (p ≤ 0.001; Figure 4A). As in skeletal muscle, glycogen content in heart followed an exponential decay curve when plotted against VCN in bone marrow. The exponential decay constant λ was seven times higher in LV-*IGF2.GAAco*-treated mice compared with LV-*GAAco* treatment (λ_LV-*IGF2.GAAco*_ = 1.141, λ_LV-*GAAco*_ = 0.159; Figure 4B; for statistical outcome see Table S2). As in skeletal muscles, full correction of glycogen content was achieved at VCN 3 after lentiviral gene therapy with LV-*IGF2.GAAco*, while, at the same, VCN LV-*GAAco* caused only a partial reduction of glycogen content (Figure 4B).

*Gaa*^−/−^ mice presented increased levels of LC3-I, LC3-II, and p62 in heart compared with WT mice, while beclin 1 levels remained unchanged (Figures 4C–4F). After gene therapy at low dose, LV-*IGF2.GAAco* and LV-*GAAco* equally reduced p62 expression to WT levels (Figure 4E). At the same low dose, LC3-I expression was normalized after LV-*IGF2.GAAco* gene therapy, but not with LV-*GAAco* (p ≤ 0.01), whereas LC3-II levels were normalized by both treatments (Figure 4F). At high dose (MOI 7, 9 Gy, 10^6^ transplanted cells), gene therapy with LV-*GAAco* also normalized LC3-I levels (Figures 4E and 4F).

Eight-month-old *Gaa*^−/−^ mice presented with cardiac cardiomegaly measured by increased heart weight (Figure 4G).^65^ Gene therapy with both LV-*IGF2.GAAco* and LV-*GAAco* prevented cardiomegaly, but this was less pronounced at low-dose gene therapy, particularly for the LV-*GAAco*-treated mice. This difference may in part be explained by the body weight differences in the two groups mentioned above.

Hearts of *Gaa*^−/−^ mice showed a prominent presence of PAS- and AP-positive cardiac fibers (Figure 4H). High-dose gene therapy (MOI 7, 9 Gy, 10^6^ transplanted cells) with either LV-*GAAco* or LV-*IGF2.GAAco* resulted in a marked reduction of reactivity for both PAS and AP, but only treatment with LV-*IGF2.GAAco* led to complete normalization of PAS- and AP-positive fibers, mirroring the biochemical findings (Figure 4H, upper row; full cardiac sections are shown in Figure S8).

We conclude that gene therapy with LV-*IGF2.GAAco* can fully prevent glycogen accumulation, autophagic defects, and cardiac cardiomegaly in *Gaa*^*−/−*^ mice.

### Full correction of glycogen accumulation in the CNS with LV-*IGF2.GAAco* lentiviral gene therapy

**Figure 5:**
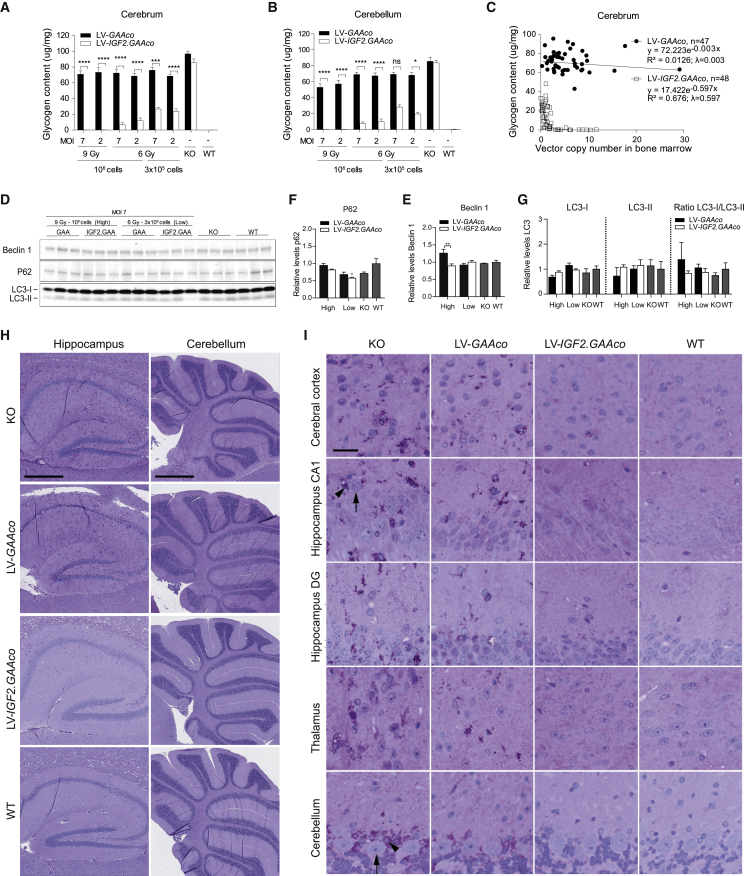
Gene therapy with LV-*IGF2.GAAco* results in correction in brain (A and B) Glycogen content in control and gene-therapy-treated mice in cerebrum and cerebellum. MOI, multiplicity of infection; Gy, gray. (C) Correlation between VCN and glycogen clearance in cerebrum. λ, exponential decay constant. VCN is not normalized for chimerism. (D–G) Immunoblot analysis for beclin 1, p62, and LC3 in cerebrum after gene therapy. Age-matched WT and *Gaa*^*−/−*^ animals were taken as control. Density levels of beclin 1 (E), p62 (F), or LC3 (G) were quantified from (D) (total protein control is shown in Figure S5C). (E–G) Quantification density values are relative to WT level (set as 1). (H and I) PAS staining analysis in cerebrum and cerebellum after high-dose gene therapy. An overview of the stained area is presented in (H). Scale bar, 0.5 mm (hippocampus) or 1 mm (cerebellum). Representative images at indicated areas are shown in (I). Scale bar, 50 μm. Black arrows point to pyramidal neurons (in hippocampus CA1) and Purkinje cells (in cerebellum). Black arrowheads refer to the glial cells. DG, dentate gyrus; KO, untreated *Gaa*^−/−^ mice; WT, untreated WT mice. Data are presented as means ± SEM. (A and B) Data are analyzed by two-way ANOVA with Bonferroni’s correction, using vector (LV*-GAAco* or LV-*IGF2.GAAco*) and gene therapy dose as nominal predictor variables. Significant results are indicated by brackets. Results of multiple comparison analysis are reported in Table S3. Western blot quantifications (E–G) are analyzed by one-way ANOVA followed by Bonferroni’s multiple testing. Significance versus WT is shown; other significant comparisons are indicated by brackets. LV-*GAAco* and LV-*IGF2.GAAco*, n = 7; KO, n = 5; and WT, n = 5. (E–G) n = 3. (H and I) LV*-GAAco* and LV-*IGF2.GAAco*, n = 3 per group; KO, n = 2; and WT, n = 2. ns, not significant; ∗p ≤ 0.05, ∗∗p < 0.01, ∗∗∗p < 0.001, ∗∗∗∗p ≤ 0 .0001.

*Gaa*^−/−^ mice showed increased glycogen levels in tissue lysates of the cerebrum and cerebellum (Figures 5A and 5B). LV-*GAAco* gene therapy reduced glycogen levels in the cerebrum by a maximum of 30%, but failed to cause a dose-dependent reduction (Figure 5A), which confirmed that glycogen levels were at most partially reduced after gene therapy with LV-*GAA* or LV-*GAAco*.^49^^,^^51^ A slightly better response to LV-*GAAco* gene therapy was observed in cerebellum, where low-dose gene therapy resulted in 20% reduction of glycogen levels, while high-dose gene therapy reduced cerebellar glycogen levels up to 40% (Figure 5B). In contrast, gene therapy with LV-*IGF2.GAAco* showed a dose-dependent effect on cerebral and cerebellar glycogen, leading to a reduction of 70% at low dose (6 Gy, 3 × 10^5^ transplanted cells), a reduction of 96% at intermediate dose (6 Gy, 10^6^ transplanted cells), and complete normalization at high-dose gene therapy (9 Gy, 10^6^ transplanted cells; Figures 5A and 5B). Glycogen content in the brain showed a significant exponential decay relationship with VCN in bone marrow after LV-*IGF2.GAAco* gene therapy (λ = 0.597), but not after LV-*GAAco* gene therapy (λ = 0.003; Figure 5C). LV-*IGF2.GAAco* caused a complete normalization of brain glycogen levels at a VCN between 0.5 and 3.

*Gaa*^−/−^ mice did not show increased expression of autophagic markers when total cerebrum extracts were analyzed, with unchanged levels of LC3, p62 and Beclin-1 compared with WT mice (Figures 5D–5G), as previously reported.^66^ Gene therapy with either vector had no effect on the levels of the autophagic components analyzed (Figures 5D–5G). PAS staining of sections from *Gaa*^−/−^ mice showed a widespread accumulation of glycogen in hippocampus and cerebellum (Figure 5H), with pronounced staining in cerebral cortex (layer III/IV), hippocampus (CA1 and dentate gyrus), thalamus, hypothalamus, midbrain, olfactory bulb and cerebellum, in agreement with previous studies (Figures 5I and S3A).^67^ Large neurons, including pyramidal neurons in the hippocampal area CA1 and cerebellar Purkinje cells (black arrows), were almost free of glycogen deposits, whereas adjacent glial cells (black arrow heads) showed strong PAS reactivity. In addition, cerebellar white matter and corpus callosum also showed PAS-positive areas (Figure 5H). At high dose, gene therapy with LV-*IGF2.GAAco* caused complete normalization of PAS reactivity in all these areas, while LV-*GAAco* had no to mild effect (Figure 5; scoring of PAS reactivity in brain is shown in Figure S3A). In conclusion, these data demonstrate that gene therapy with LV-*IGF2.GAAco* was able to prevent glycogen accumulation in the cerebral cortex, hippocampus, thalamus, and cerebellum almost completely.

### Robust alleviation of neuroinflammation by LV*-IGF2.GAAco* lentiviral gene therapy

**Figure 6:**
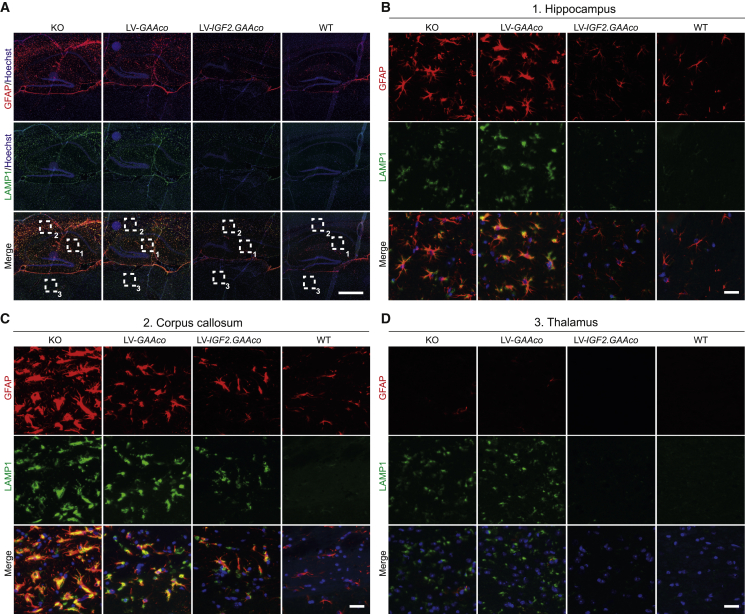
Gene therapy with LV-*IGF2.GAAco* relieves astrogliosis in brain (A–D) Sagittal sections of hippocampus, corpus callosum, and thalamus after high-dose gene therapy stained for GFAP (red) and LAMP1 (green). An overview of the stained area is presented in (A). Scale bar, 500 μm. Boxed areas in (A) are magnified in (B) (1, hippocampus), (C) (2, corpus callosum), and (D) (3, thalamus). Scale bar, 25 μm.

Neuroinflammation, characterized by astrocyte and microglial activation, is a common characteristic of CNS pathology in several LSDs.^68^ By staining astrocytes for glial fibrillary acid protein (GFAP), we observed regional upregulation of GFAP expression in the brain of KO mice, which was most prominent in the hippocampus (Figures 6A and 6B) and corpus callosum (Figures 6A and 6C), but not in the thalamus (Figure 6D). In addition, GFAP-positive cells showed a prominent thickening of the cell body and processes, a typical morphological change of reactive astrocytes.^69^^,^^70^ Besides increased GFAP reactivity, *Gaa*^−/−^ mice presented increased lysosomal size in the hippocampus, corpus callosum, and thalamus, as visualized by staining for lysosomal associated membrane protein 1 (LAMP1; Figures 6A–6D and 6E–6H). Importantly, LAMP1 colocalized with GFAP in hippocampus and corpus callosum, suggesting that astrocytes are severely affected in these areas (Figures 6B and 6C). Upon treatment with high-dose gene therapy, only LV-*IGF2.GAAco* reduced GFAP and LAMP1 immunoreactivity, with a more prominent effect in hippocampus (complete normalization) compared with corpus callosum (partial reduction), while LV-*GAAco* gene therapy had no effect in any of these areas (Figures 6A–6D).

**Figure 7:**
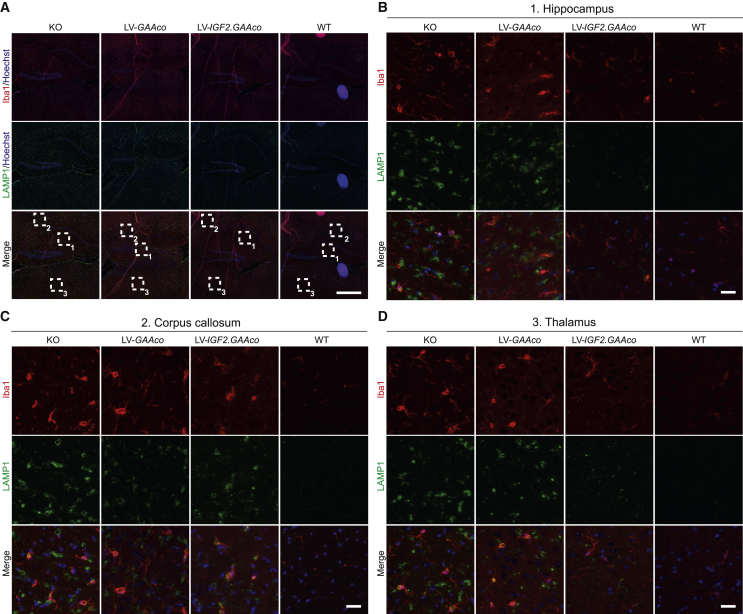
Gene therapy with LV-*IGF2.GAAco* relieves microgliosis in brain (A–D) Sagittal sections of hippocampus, corpus callosum and thalamus after high-dose gene therapy stained with Iba1 (red) and LAMP1 (green). An overview of the stained area is presented in (A). Scale bar, 500 μm. Boxed areas in (A) are magnified in (B) (1, hippocampus), (C) (2, corpus callosum), and (D) (3, thalamus). Scale bar, 25 μm.

We also investigated ionized calcium-binding adapter molecule 1 (Iba1) expression to assess microglial activation. Unlike the regional activation of astrocytes, a more widespread increase of Iba1 immunoreactivity was observed in *Gaa*^−/−^ brain (Figure 7A, overview); i.e., in hippocampus (Figure 7B), corpus callosum (Figure 7C), and thalamus (Figure 7D). All the areas analyzed showed a distinctive morphological switch of IBA1-positive cells from a ramified shape in WT mice to a more amoeboid appearance in KO mice, a typical transformation of microglia upon activation.^71^^,^^72^ LAMP1 colocalized with IBA1 in all the areas, suggesting lysosomal swelling in microglial cells (Figures 7A–7D). After high-dose gene therapy with LV-*IGF2.GAAco*, microglial activation was barely detectable in in the brain, including hippocampus (Figure 7B) and thalamus (Figure 7D), except for the corpus callosum where microglial activation was still present to some extent (Figure 7C). In contrast, widespread microglial activation was still present after high-dose gene therapy with LV-*GAAco*.

In conclusion, we demonstrated that *Gaa*^*−/−*^ mice have prominent neuroinflammation, shown by the activation of both astrocytes and microglia. Gene therapy using LV-*IGF2.GAAco* but not LV-*GAAco* was able to normalize or strongly reduce neuroinflammation and lysosomal pathology.

### Gene therapy with LV-*IGF2.GAAco* does not affect blood glucose levels

**Figure 8:**
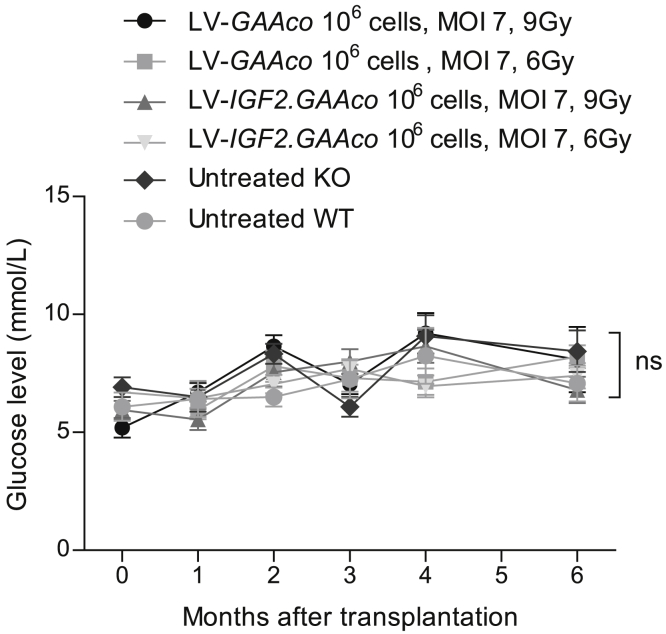
Glucose levels are not affected by gene therapy with LV-*IGF2.GAAco* Monthly glucose levels in plasma of mice treated as indicated. Mice were fasted for 15 h before glucose sampling. Data are presented as means ± SEM and analyzed by ANOVA with *post hoc* Turkey’s analysis. n = 10 per gene-therapy-treated groups; n = 6 per control groups; ns, not significant.

Recent clinical trials showed transient hypoglycemia in patients with Pompe disease shortly after intravenous injection of recombinant IGF2.GAA at 10–20 mg/kg, but not at 5 mg/kg.^73^ To investigate whether long-term exposure to IGF2.GAA protein after gene therapy interferes with blood glucose levels, we monitored glucose levels monthly in mice treated with two different doses of LV-*IGF2.GAAco* gene therapy, the highest dose (MOI 7, 9 Gy, 10^6^ transplanted cells) or a lower dose (MOI 7, 10^6^ transplanted cells, 6 Gy). Over the course of the experiment of up to 6 months after transplantation, we observed significant differences in glucose levels neither in untreated KO and WT mice nor in KO mice treated with LV-*IGF2.GAAco* or LV-*GAAco* gene therapy at any dose or time point (Figure 8).

GAA activity levels in plasma were compared after gene therapy and shortly after intravenous injection of rhGAA (Figure S9). Plasma GAA activity in animals treated with high-dose LV-*GAAco* or LV-*IGF2.GAAco* reached levels of 850 nmol/h/mL (∼0.023 mg/mL) and 188 nmol/h/mL (∼0.005 mg/mL), respectively (Figure S9). In contrast, intravenous injection of 20 mg/kg rhGAA resulted in plasma GAA activity of more than 40,000 nmol/h/mL (∼1 mg/mL) within 5 min post injection. This shows that gene therapy with LV-*IGF2.GAAco* results in plasma GAA levels that are more than 200-fold lower than those reached by intravenous injection of rhGAA at 20 mg/kg.

## Discussion

ERT has improved the life expectancy and motor outcome of infants with classic infantile Pompe disease significantly, but it becomes more and more evident that this treatment does not provide a cure. Limitations of the therapy are its variable efficacy and its inability to pass the BBB. Previously, we reported that in a mouse model for Pompe disease, lentiviral gene therapy with LV-*GAA* or LV-*GAAco* reduced glycogen levels in most of the tissues, but one of the limitations was that high vector doses were needed and that glycogen was not fully cleared from the major target tissues, especially not from the brain.^49^^,^^51^ This was a reason for continuing our efforts to identify a lentiviral vector with improved efficacy. In the present study, we investigated the therapeutic efficacy of HSPC-mediated gene therapy using LV-*IGF2.GAAco*. We found that LV-*IGF2.GAAco* was able to fully normalize histopathology, impairment of autophagy, cardiomegaly, and motor function. In the brain, LV-*IGF2.GAAco* prevented glycogen accumulation and neuroinflammation. These results demonstrate that HSPC-mediated lentiviral gene therapy using LV-*IGF2.GAAco* is able to achieve full correction of glycogen accumulation and pathology in murine Pompe disease.

### Brain pathology

The CNS has emerged as a novel target for therapy for Pompe disease in recent years with the finding that classic infantile patients progressively develop white matter abnormalities and impaired performance in neuropsychological tests.^25^, ^26^, ^27^^,^^30^^,^^31^ CNS pathology has also been demonstrated by autopsies of classic infantile patients showing glycogen accumulation and regional gliosis.^74^, ^75^, ^76^, ^77^, ^78^, ^79^, ^80^, ^81^, ^82^ Glycogen accumulation has also been observed in the brain of different mouse models of Pompe disease.^49^^,^^67^ We found that *Gaa*^−/−^ mice at 8 months of age exhibited widespread glycogen accumulation that was most pronounced in glial cells in the cortex, hippocampus, thalamus, and cerebellum, in line with results found using the neo6/neo6 *Gaa*^−/−^ mouse model.^67^

In addition, we found evidence of strong neuroinflammation by immunofluorescent analysis of microglia and astrocytes in brain sections. In agreement, Yambire et al.^83^ also found increased numbers of astrocytes at as early as 6 months of age in the cortex of neo6/neo6 *Gaa*^−/−^ mice using GFAP staining. Using the same mouse model, Sidman et al.^67^ found evidence of astrogliosis only at the oldest ages examined (15 and 22 months). The different observations may be explained by differences in the immunofluorescent protocols employed in these two studies. Besides astrogliosis in the cortex, we found a widespread increase in astrocyte cell numbers, which was accompanied by a widespread activation of microglia throughout the CNS. This resembles findings from autopsies of classic infantile Pompe patients. Several of these studies reported widespread gliosis as well as neuronal loss.^77^, ^78^, ^79^^,^^81^^,^^82^ The CNS abnormalities observed in Pompe disease compare well with the findings in CNS found in other LSDs affecting the brain. These studies also displayed neuroinflammation reflected by activated microglia and astrogliosis.^68^

### Correction of brain pathology by lentiviral gene therapy

HSPC-LVGT expressing arylsulfatase A (ARSA) has been shown to improve CNS pathology for MLD, another lysosomal storage disease.^40^^,^^46^^,^^47^ Studies in a mouse model showed that the underlying mechanism involved preconditioning-induced damage to the BBB, followed by migration of gene-corrected HSPC-derived monocytes across the BBB, and differentiation of monocytes into microglial-like cells.^84^, ^85^, ^86^ A clinical trial with LV gene therapy for MLD was started 8 years ago and the results demonstrated strong improvement of the CNS phenotype.^46^^,^^47^ Our previous results indicated that HSPC-LVGT employing native GAA failed to correct glycogen accumulation in the brain, while GAAco led to a reduction of cerebral glycogen levels at high VCN.^49^^,^^51^ In the present study, a dose-response analysis was performed comparing an untagged GAAco transgene^51^ with IGF2-tagged GAAco (this study). Untagged GAAco largely failed to completely normalize brain glycogen levels under the conditions employed, whereas IGF2 tagged GAAco fully normalized brain glycogen at a clinically relevant VCN. Correction of glycogen in the cerebellum by IGF2.GAA at low gene therapy doses might explain the improved performance in the rotarod. This indicated that epitope tagging with IGF2 provided strong improvement of lentiviral gene therapy for correcting murine Pompe disease at a dose suitable for clinical applications. We hypothesize that the effect of the IGF2 epitope was mediated by enhanced cross correction of cells in the CNS parenchyma. However, additional mechanisms cannot be ruled out; e.g., transcytosis of IGF2.GAA protein through the BBB that can act as a synergistic mechanism. Because it is not restricted to non-dividing cells and because it can treat the CNS, LVGT would be applicable to a clinical translation for all the forms of Pompe disease and especially for the classic infantile Pompe disease patients. Both these aspects are limitations of AAV-based gene therapy approaches.

### Correction of muscle pathology by LV-*IGF2.GAAco*

We found a lack of correlation between GAA activity in lysates and normalization of glycogen levels (Table 2). We confirmed this result by performing immunoblot analysis using a human GAA antibody (Figure S4). We found that the apparent molecular weight of the 76-kDa active form of GAA was slightly higher in tissues treated with LV-*GAAco* compared with LV-*IGF2.GAAco*. Whether this relates to differential intracellular processing and/or post-translational modification and/or ability to restore autophagic flux (Figures 3 and 4) should be investigated in future work. A discrepancy between tissue GAA enzymatic activity and normalization of glycogen levels has also been found previously by another study.^58^ Here, rhGAA (myozyme) and IGF2-tagged rhGAA proteins were administered to GAA knockout mice by four weekly intravenous injections. Although IGF2.GAA was superior in reducing glycogen levels compared with GAA, total GAA activities in total tissue lysates were lower in IGF2.GAA compared with GAA-treated mice. It has been hypothesized that this might be caused by preferential uptake of enzyme by unrelated cells that are present in the tissues, such as endothelial cells.^58^

Enzyme levels in total tissue homogenate do not distinguish between inter- and intracellular distribution of the GAA enzyme. In total tissue homogenates, GAA protein could be present in skeletal muscle cells as well as fibroblasts or endothelial cells. In line with this hypothesis, Fukuda and colleagues showed that GAA activity levels in total tissue homogenates of rhGAA-treated Pompe mice do not correlate with GAA activity levels in isolated muscle fibers.^87^ In addition, it is known that in Pompe disease autophagy is blocked, and that, as a result, GAA trafficking toward lysosomes is affected by impaired autophagy: a significant portion of the endocytosed enzyme was shown to be trapped in autophagic areas rather than reaching the lysosomes and clearing lysosomal glycogen accumulation.^87^, ^88^, ^89^, ^90^, ^91^ Fukuda and colleagues also showed impaired processing of the GAA protein in isolated muscle fibers, but not in total tissue homogenates.^87^ This supports the hypothesis that high GAA activity levels in muscle tissue homogenates do not necessarily translate into high levels of GAA enzyme in skeletal muscle cells and/or in lysosomes and therefore do not necessarily correlate with clearance of glycogen accumulation in muscle fibers. It is therefore difficult to reliably correlate GAA enzyme activity in total tissue homogenates with glycogen levels in tissue extracts.

Previous findings from our laboratory have also suggested a poor correlation between muscle rhGAA enzyme activity values and restoration of muscle pathology in biopsies from patients treated with ERT. This is possibly due to accumulation of intravenously administered rhGAA in the interstitium, but it could also be explained by individual variability, sampling variability, or possibly by the poor uptake of rhGAA by skeletal muscle tissues. Clinical studies on the effects of ERT have indeed used glycogen levels rather than GAA enzyme activity in muscle biopsies as outcome measures.^92^ In a mouse model for another lysosomal disease, MPS II, treatment with HSPC-LVGT expressing iduronate sulfatase (IDS) or epitope tagged IDS (IDS.ApoE2) also resulted in a lack of correlation between IDS enzyme activity in tissue lysates and in clearance of glycosaminoglycans in the brain.^93^ Therefore, a limitation of measuring enzyme activity in tissue lysates is that it does not reflect where the enzyme activity is located. In fact, the enzyme activity might result from enzyme present outside or in other cells than the target cells that are most in need of enzyme correction.

### Safety of HSPC-LVGT with LV-*IGF2.GAAco* with respect to genotoxicity and glucose homeostasis

Lentiviral transduction using third-generation vectors has proved to be a safe approach without causing detrimental genotoxicity events in a number of clinical trials to date, including MLD, X-linked adrenoleukodystrophy, Wiskott-Aldrich syndrome and X-linked severe combined immunodeficiency (SCID), which are ongoing with a current maximum median follow-up of more than 7 years. All these trials limited the number of vector copies to four per diploid genome, which is within the therapeutic VCN dose found in our studies.^42^^,^^45^, ^46^, ^47^^,^^52^^,^^54^, ^55^, ^56^ Integration site analysis in clinical trials for MLD and Wiskott-Aldrich syndrome demonstrated the relatively safe integration profile of the lentiviral vectors and a polyclonal reconstitution of the hematopoietic system by HSPCs.^47^^,^^56^^,^^94^ However, in a recent phase 1 trial with the globin vector TNS9.3.55 for transfusion-dependent thalassemia (TDT), all four patients developed clonal hematopoiesis associated with transactivation of cancer-related genes after HSPC-mediated lentiviral gene therapy, although all cases appeared to be benign so far (NCT01639690; median VCN in blood was 0.03; follow-up of 6–8 years).^95^ Other two phase 1/2 trials with a different globin vector, BB305, for TDT and sickle cell disease (SCD) did not find clonal expansion associated with insertional mutagenesis for any of the 22 patients involved in the study (HGB-204/HGB-205; median VCN in blood 0.95; follow-up of 4.6–7.9 years).^44^^,^^96^, ^97^, ^98^, ^99^ Vectors BB305 and TNS9.3.55 show an overall similar architecture, but they contain different segments of the β-globin promoter and locus control region (LCR), which regulate the expression of the human β-globin gene. The choice of promoter/LCR segments is likely to have played a role by conferring a transactivation activity on neighboring genes. These results stress the importance of promoter choice and the viral dosage when it comes to clinical implementation.^100^ It will be important to carefully consider these aspects and to perform the available *in vitro* genotoxicity tests of the clinical lentiviral vector prior to use in patients. In addition, it will be important to clearly disclose the possible risk of the treatment to patients and their parents. The SF promoter employed in the present study is not considered to be a safe option for clinical development. To address this, we have tested alternative promoters. This identified the clinically acceptable MND promoter^59^, ^60^, ^61^ to have the same efficacy as the SF promoter (Figure S10), indicating that a lentiviral vector expressing the *IGF2*.*GAA* transgene under the control of the MND promoter may well be suitable for clinical translation.

The preconditioning regimen performed prior to transplantation is pivotal to ensure an efficient and long-lasting engraftment of HSPCs. Busulfan- or treosulfan-based regimens have been shown to be immunosuppressive and myeloablative, and have a similar ablative nature compared with the non-clinically relevant irradiation.^101^ Future experiments with clinically relevant preconditioning regimens are therefore required to confirm the *bona fide* translation of our approach to clinical settings. Our results demonstrate that correction of Pompe disease pathology correlates with the strength of the preconditioning administrated prior to transplantation to maximize chimerism and thereby expression of the *GAAco* and *IGF2.GAAco* transgenes. Over the years, preconditioning regimens such as busulfan and treosulfan have been used in clinical trials for lentivector-based gene therapies and have proved to be relatively safe even when a full myeloablative dose is administrated.^95^^,^^96^^,^^102^, ^103^, ^104^, ^105^

Intravenously applied ERT using IGF2.GAA has been tested in a clinical trial with adult-onset Pompe patients, and this revealed a dose-dependent transient induction of hypoglycemia starting at a dose of 10 mg/kg during and/or within 2 h of the end of infusion.^73^ At 5 mg/kg, no hypoglycemia was observed. We found that GAA and IGF2.GAA activity levels in plasma after high-dose LVGT were 50–200 times lower compared with bolus ERT infusion of Myozyme (20 mg/kg), and that blood glucose levels remained unaffected. This indicates that, in the mouse, therapeutic levels of IGF2.GAA were several orders of magnitude below the levels that can cause hypoglycemia. However, this encouraging result does not alleviate the need for carefully testing possible effects of IGF2.GAA on glucose homeostasis in human patients in a future clinical trial.

## Materials and methods

### Animals and procedures

*Gaa* knockout (*Gaa*^−/−^) mice contained a targeted disruption of exon 13 and have been described previously by us.^65^ Mice were maintained in the FVB/n background. This model reflects human Pompe disease caused by deficiency of GAA, resulting in generalized glycogen storage in various tissues, including skeletal muscle, the heart, and the brain.^65^^,^^106^, ^107^, ^108^ Age-matched FVB/n mice were obtained from Charles River as WT controls. All mice were housed under specific pathogen-free (SPF) conditions in the Laboratory Animal Science Center (EDC) at the Erasmus MC and bred according to standard procedures, which included a 12-h light-dark cycle and *ad libitum* diet. Body weight was determined before sacrifice and we noticed that body weight at sacrifice (at 8 months) is influenced to a large extent by the body weight at the beginning of the experiment (2 months of age) and that this is mainly dependent on random variation. Mice were fasted 15 h pre-sacrifice to deplete cytoplasmic glycogen.^107^ Subsequently, mice were anesthetized by ketamine (10%, Alfasan, Woerden, the Netherlands) and Sedator (1 mg/mL, Eurovet, Bladel, the Netherlands) and sacrificed by either intracardiac perfusion with phosphate-buffered saline (PBS), PBS followed by paraformaldehyde (PFA), or by cervical dislocation. Relevant tissues were harvested, snap-frozen in liquid nitrogen, and stored at −80°C until further analysis. All animal experiments in this study were approved by the Animal Experiments Committee (DEC) in the Netherlands and these complied with the Dutch legislature to use animals for scientific procedures.

### Lentiviral vector construction and production

Codon-optimized human *GAA* (*GAAco*; GenScript, Piscataway, NJ) was cloned into the third-generation self-inactivating (SIN) lentiviral vector pRRL.PPT.SF.GFP.bPRE4∗.SIN (LV-*SF-GFP*^49^) by replacing the *GFP* gene using AgeI and SbfI restriction sites to generate pRRL.PPT.SF.GAAco.bPRE4∗.SIN (LV-*GAAco*).^51^ A codon-optimized insulin-like growth factor 2 (IGF2) cassette (GenScript, Piscataway, NJ) was subcloned into the LV-*GAAco* backbone after double digestion using BamHI and SgrAI. The resultant lentiviral vector pRRL.PPT.SF.IGF2.GAAco.bPRE4∗.SIN (LV-*IGF2.GAAco*) encodes the IGF2 signal peptide, residues 1 and 8–67 of human IGF2, a three-amino-acid spacer, and residues 70–952 of codon-optimized human GAA (Figure 1A).^58^ Transgene expression was driven by the spleen focus-forming virus (SFFV) promoter. The SFFV promoter was substituted with the MND promoter (myeloproliferative sarcoma virus enhancer, negative control region deleted, dl587rev primer-binding site substituted) through XhoI/AgeI restriction sites, thus generating the pRRL.PPT.MND.GAAco.bPRE4∗.SIN (MND-LV-*GAAco*). Lentivirus was generated in HEK 293T cells by calcium phosphate transfection with the third-generation lentiviral vector packaging plasmids pMDL-g/pRRE, pMD2-VSVg, and pRSV-Rev.^109^^,^^110^ Virus concentration was performed by ultracentrifugation (Beckman, SW32Ti rotor) at 20,000 rpm for 2 h at 4°C ,and titration was performed by quantitative polymerase chain reaction (qPCR) with primers targeting the U3 and Psi sequences of *HIV* (listed in Table S1). A standard curve was prepared using transduced HeLa with on average one copy of integrated lentiviral vector per genome. Final titers were determined as the average VCNs multiplied by the cell number and fold dilution. Viral batches were prepared, analyzed, and quantified side by side, routinely obtaining titers of 10^8^ infectious units/mL for all viral vector lots.

### Secretion and uptake of GAA and IGF2.GAA *in vitro*

HEK 293T cells were grown in Ham’s F-10 medium (Lonza) supplemented with 10% fetal bovine serum (FBS) (Biowest) and 1% penicillin-streptomycin (PS) (Gibco) and transduced with LV-*GAAco* or LV-*IGF2.GAAco* at an MOI of 10. Cells were harvested 5 days post transduction and vials were frozen as IGF2.GAA or GAA protein producer cell lines for subsequent *in vitro* assays. For production of conditioned media, HEK 293T producer cells were grown at 90% confluency in Ham’s F-10 medium supplemented with 10% FBS, 1% PS, and 3 mM PIPES (Sigma). After 24 h, cells and media were collected. Conditioned medium containing secreted IGF2.GAA or GAA was filtered (0.22-μm filter, Millipore) and used for uptake assays. Where indicated, conditioned medium was supplemented with M6P (Sigma, #M3655) or IGF2 (Cell Sciences, #MU100) for competitive inhibition. Uptake experiments were performed on primary myoblasts isolated from *Gaa*^−/−^ mice as previously described.^111^ Myoblasts were cultured in growth medium (1% PS and 20% FBS [Biowest] in Ham’s F-10 medium [Lonza] to 90% confluency on extracellular matrix [ECM]-coated plates [Sigma, 5%] and differentiated into myotubes in differentiation medium [1% PS and 2% horse serum; Gibco] in high-glucose Dulbecco’s modified Eagle’s medium [DMEM, Lonza]) at 37°C with 5% CO_2_. Enzyme activity was determined in conditioned medium collected from HEK 293T cells (described above), and GAA protein with an activity of 800 nmol/h/mL was incubated on myotubes for 24 h rhGAA (Myozyme, Genzyme Corporation) was used as positive control. Media and cells were harvested after 24 h and GAA enzyme assay and western blotting were performed. For analysis of secretion, HEK 293T cells were transfected as previously shown by Bergsma et al.^112^ In short, GAAco or IGF2.GAAco cDNAs were cloned into pcDNA3.1 expression vector using XhoI/XbaI restriction enzymes and transfected into HEK 293T cells. Two-hundred milliliters of medium were sampled every 24 h and up until 96 h post transfection for GAA enzyme activity analysis. At 96 h, cells were washed with PBS and lysed as described below. GAA activity was measured in medium samples and cell lysate using 4 MU analysis as described below. Western blot analysis was performed on cell lysate and medium samples at 96 h post transfection as described below. Transfection efficiency was measured based on mRNA expression of the Neomycine resistance cassette present in the pcDNA3.1 backbone using RT-qPCR, as previously described.^112^

### Western blotting

Protein extracts from cells were obtained in lysis buffer (100 mM NaCl, 50 mM Tris [pH 7.5], 1% Triton X-100) supplemented with protease inhibitors (Complete Protease Inhibitor Cocktail, Roche) and phosphatase inhibitors (50 mM NaF). Tissue samples were homogenized in RIPA buffer (150 mM NaCl, 50 mM Tris (pH 7.5), 1% Triton X-100, 0.1% SDS, 0.5% sodium deoxycholate) supplemented with protease inhibitors (Complete Protease Inhibitor Cocktail, Roche) and phosphatase inhibitors (50 mM NaF) using 5-mm stainless steel beads (Qiagen) in a TissueLyser II (Qiagen, Venlo, the Netherlands) for 5 min at 30 Hz. Debris was pelleted by centrifugation at 10,000 rpm for 5 min. Supernatant was incubated overnight with Benzonase Nuclease (10 units/100 μL lysate; Sigma-Aldrich, St. Louis, MO; #E1014) at 4°C. Protein concentration was determined using a Pierce BCA Protein Assay Kit (Thermo Fisher Scientific) according to the manufacturer’s instructions. Fifty micrograms (gastrocnemius, heart, and tibialis anterior) or 20 μg (diaphragm and quadriceps femoris) of total protein were used for GAA protein analysis in skeletal muscles and heart. To highlight differences in the apparent molecular weight of GAA protein, 10 times less total protein from LV-*GAA*-treated mice was used for immunoblots in Figures S4J and S4K. A total of 20 μg (tibialis anterior and brain) or 30 μg of protein (heart) were used for autophagy analysis in tissues. Samples were denatured with 5× Laemmli sample buffer (62.5 mM Tris-HCL pH 6.8, 2% SDS, 25% glycerol, 0.01% bromophenol blue, 5% β-mercaptoethanol) and heated at 95°C for 5 min. Protein extracts of cell lysates were separated by SDS-PAGE on a 4%–15% polyacrylamide gel (Criterion TGX, Bio-Rad) and total protein load was measured using a Geldoc XR+ (Bio-Rad). Proteins were transferred to nitrocellulose blotting membranes (GE Healthcare) and blocked with 5% non-fat milk powder in PBS or Tris-buffered saline (TBS) and probed by overnight incubation at 4°C with rabbit anti-GAA (1:1,000, Abcam, clone EPR4716(2)) and mouse anti-GAPDH (1:1,000, Millipore) or with rabbit anti-SQSTM/P62 (1:1,000, Cell Signaling Technology, #5114), rabbit anti-Beclin 1 (D40C5) (1:1,000, Cell Signaling Technology, #3495), or rabbit anti-LC3 (1:1,000, Cell Signaling Technology, #2775) in 5% non-fat milk powder in PBS supplemented with 0.1% Tween. Proteins of interest were detected with IRDye 800 CW and IRDye 680 RD secondary antibodies (1:10,000 to detect anti-GAA and anti-GAPDH; 1:5,000 for all other antibodies; LI-COR Biosciences, Lincoln, NE) and were imaged using the Odyssey Infrared Imaging System (LI-COR Biosciences, Lincoln, NE). Protein content was quantified using Fiji; in the autophagy blots, equal loading was determined by quantification of the total bands using the stain-free signal on the same gel used for immunoblotting.

### Lentiviral HSPC transduction and transplantation procedures

HSPC-LVGT was conducted in two large experiments, one using the LV-*GAAco* vector and another the LV-*IGF2.GAAco* vector. Untreated *Gaa*^−/−^ and FVB/N WT mice were included in each round as internal controls. The experiments were performed by the same investigator (Q.L.) within 2 weeks using identical procedures. Bone marrow cells were harvested from 8-week-old male *Gaa*^−/−^ mice and hematopoietic stem and progenitor cells were enriched by lineage depletion (Lin^−^) using the Mouse Hematopoietic Progenitor Cell Enrichment Set (BD Sciences, San Jose, CA). Lin^−^ cells were seeded at a density of 10^6^ cells/mL in StemMACS HSPC expansion medium (Miltenyi Biotec, Leiden, the Netherlands), supplemented with murine thrombopoietin (100 ng/mL), murine stem cell factor (100 ng/mL), and human FMS-like tyrosine kinase 3 murine ligand (50 ng/mL).^49^ Different vector dosage (MOI), number of transplanted cells, and irradiation doses were administrated as detailed in Table 1. Cells were transduced overnight at MOI 7 or 2 with concentrated lentiviral particles and incubated at 37°C with 10% CO_2_. The following day, 5 × 10^5^ or 10^6^ transduced Lin^−^ cells were transplanted intravenously into 8-week-old female *Gaa*^−/−^ recipients, previously subjected to 6 or 9 Gy of total body irradiation (TBI) using the Gammacell 40 irradiator (Atomic Energy of Canada, ON, Canada). The transplantation procedure for the comparison of the SF and the MND promoters was performed using a low-dose gene therapy with MOI 7, 10^6^ transduced Lin^−^ cells, and 6 Gy of TBI to allow the detection of differences in the outcome of the treatment. No normalization for body weight was applied to the number of cells transplanted.

### Rotarod

Motor function was determined for 8-month-old mice on an accelerating rotarod, accelerating from 4 to 40 rpm in 5 min (Panlab, Harvard Apparatus, Holliston, MA).^49^ Each mouse was tested three times with intervals of 5 min. Latency was expressed as average of the three tests.

### GAA enzymatic assay and glycogen content measurements

Bone marrow and leukocytes were lysed in water supplemented with protease inhibitors (cOmplete, Roche) by three freeze-thaw cycles. Cell pellets from *in vitro* experiments were lysed in lysis buffer supplemented with protease inhibitors (cOmplete, Roche). Tissue samples were homogenized in water by TissueLyser II (Qiagen, Venlo, the Netherlands) at 30 Hz for 5 min and debris was pelleted by centrifugation for 10 min at 10,000 rpm. GAA activity was measured in the supernatant using 4-methylumbelliferyl-α-D-glucoside (2.2 mM, Sigma-Aldrich, St. Louis, MO) as substrate.^113^ Glycogen was quantified by measuring the amount of glucose after conversion by amyloglucosidase and amylase (Roche Diagnostics, Basel, Switzerland) as previously described,^107^ and products were measured on a Varioskan at 414 nm (Thermo Scientific, Waltham, MA). Results from GAA and glycogen assays were normalized for protein content using the Pierce BCA protein assay kit (Thermo Scientific, Waltham, MA).

### Histopathology and immunofluorescence

PAS staining was performed on tissue fixed in glutaraldehyde and processed in paraffin (heart and tibialis anterior) or glycol methacrylate (GMA)-embedding medium (brain), and sectioned at 4 μm according to a standard protocol.^65^^,^^114^ Scoring of PAS reactivity in brain was adapted based on a method previously described for skeletal muscle.^114^^,^^115^ The level of PAS reactivity and vacuolization was determined on a scale from 1 (no staining) to 6 (very strong staining and vacuolization throughout the field) by two independent operators blinded to the experimental and control groups. The scale system is described in Figure S3B. AP staining was performed on 8- to 10-μm-thick cryosections from tissue embedded in Tissue-Tek O.C.T. Compound (Tissue-Tek, Sakura Finetek) frozen in liquid nitrogen with an isopentane interphase. Sections were scanned by a NanoZoomer 2.0 (Hamamatsu Photonics, Japan). Immunofluorescent stainings were performed on PBS- and PFA-perfused brain samples subsequently fixed in 4% PFA in PBS for 5 h, equilibrated in 20% sucrose PBS at 4°C overnight, embedded in Tissue-Tek O.C.T. Compound (Tissue-Tek, Sakura Finetek) and frozen in liquid nitrogen with an isopentane interphase. Sagittal cryostat sections (10 μm) were permeabilized with ice-cold methanol/acetone (4:1, v/v) for 10 min and blocked with 3% BSA and 0.1% Tween diluted in PBS for 30 min at room temperature. Sections were stained with primary antibodies detecting astrocytes (mouse anti-GFAP immunoglobulin [Ig] G conjugated to Cy3, 1:300, AB5804 Sigma-Aldrich), or microglia (rabbit anti-Iba1 IgG, 1:500, 019–19741 Wako Chemicals) and were co-stained with rat anti-LAMP1 IgG (clone 1D4B,1:500, Abcam). After incubation overnight at 4°C, sections were washed with PBS and labeled with the appropriate secondary antibody conjugated to Alexa Fluor 488 or Alexa Fluor 594 (1:500, Thermo Fisher Scientific) for 30 min. All sections were counterstained with Hoechst 33258 (1:15,000, Life Technologies) to stain nuclei. Pictures were obtained using an LSM 700 confocal microscope (Zeiss) with a 20× objective and analyzed by Adobe Photoshop CS6.

### Quantitative polymerase chain reaction of VCN

VCN and chimerism in bone marrow were determined by quantitative polymerase chain reaction (qPCR). Genomic DNA was extracted from bone marrow with the NucleoSpin Tissue kit (Macherey-Nagel, Düren, Germany), and used at 100 ng per qPCR using iTaq Universal SYBR Green Supermix (Bio-Rad, Hercules, CA). VCN was determined using primers specific for *HIV* (binding to U3 and Psi sequences respectively) and using a standard curve with genomic DNA from transduced mouse 3T3 cells carrying one copy of integrated lentiviral vector per genome. VCN was not normalized for chimerism. Chimerism was determined using primers specific for the *Sry* locus on the mouse Y chromosome. Both VCN and chimerism were normalized using mouse *Gapdh*. Bone marrow DNA from untreated male *Gaa*^−/−^ donor mice was used to establish a reference standard in *Sry* and *Gapdh* qPCRs. Reactions were performed in a CFX96 real-time PCR detection system and analyzed by CFX Manager 3.0 (Bio-Rad, Hercules, CA). Primer sequences are shown in Table S1.

### Glucose measurements

Plasma was collected from mice subjected to overnight fasting per time point (15 h). Glucose levels were evaluated using a Cobas C311 chemistry analyzer (Roche/Hitachi) according to the manufacturer’s protocol in the Department of Clinical Chemistry of Erasmus MC University Medical Center.

### Statistics

Statistical analysis was performed with SPSS (IBM, version 22) or GraphPad Prism (version 9.0.0. for Windows, San Diego, CA, United States, www.graphpad.com). All results are presented as mean ± SEM. Normality and lognormality tests were performed by Shapiro-Wilk test. Mann-Whitney U test was used for comparing two groups. Multiple comparison analysis was performed by one-way ANOVA with Bonferroni’s correction. Glycogen, VCN, and chimerism data are analyzed by two-way ANOVA with Bonferroni’s correction using vector type (LV-*GAAco* or LV-*IGF2.GAAco*) and gene therapy dose (combinations of 9- or 6-Gy irradiation dose, 10^6^ or 3 × 10^5^ transplanted cells and MOI 2 or 7) as categorical variables. Glycogen data for comparison of promoters (Figure S10) were analyzed by two-way ANOVA followed by Bonferroni’s multiple testing correction, using promoter (MND or SF) and skeletal muscle analyzed as categorical variables. PAS staining scoring data (Figure S3) were analyzed by two-way ANOVA followed by Bonferroni’s multiple testing correction, using treatment (LV-*GAAco* or LV-*IGF2.GAAco*) and brain area as categorical variables. Glycogen data were log2 transformed before statistical analysis. Repeated measures ANOVA with Tukey’s comparison test was used to detect differences of glucose levels between treatments over time. A p value ≤0.05 was considered statistically significant.

An exponential regression model was used to describe the relation between the VCN in bone marrow and glycogen clearance. This model is described by an exponential decay function (A0 + group × A1) × EXP((B0 + B1 × group) × VCN) where A0 denotes the initial amount for the LV-*GAAco* group, A1 denotes the difference in the initial amount between LV-*IGF2.GAAco* and LV-*GAAco*, B0 defines the exponential decay rate (λ) for group LV-*GAAco*, and B1 denotes the difference in the exponential decay rate between LV-*IGF2.GAAco* and LV-*GAAco*. To determine whether there is a difference in the exponential curves between the LV-*GAAco*- and LV-*IGF2.GAAco*-treated groups, we allowed B and A to be group dependent. When 95% confidence interval (CI) for estimated value of B1 does not contain zero, the decay in two groups is defined statistically differently. Estimated value of B1 and its 95% CI are listed in Table S2.

### Data availability

Data are available on request.

## Supplemental information

Document S1. Figures S1–S11 and Tables S1–S3

Document S2. Article plus supplemental inormation
