# Supplementary material for: IGF2-tagging of GAA promotes full correction of murine Pompe disease at a clinically relevant dosage of lentiviral gene therapy
Source: Mol Ther Methods Clin Dev. 2022 Sep 24;27:109–30. doi: 10.1016/j.omtm.2022.09.010 (PMC9573825; doi:10.1016/j.omtm.2022.09.010)
Supplement: Document S1. Figures S1–S11 and Tables S1–S3 [file mmc1.pdf]

OMTM, Volume *27*

Supplemental information

IGF2-tagging of GAA promotes full correction of murine Pompe disease at a clinically relevant dosage of lentiviral gene therapy

Qiushi Liang, Fabio Catalano, Eva C. Vlaar, Joon M. Pijnenburg, Merel Stok, Yvette van Helsdingen, Arnold G. Vulto, Ans T. van der Ploeg, Niek P. van Til, and W.W.M. Pim Pijnappel

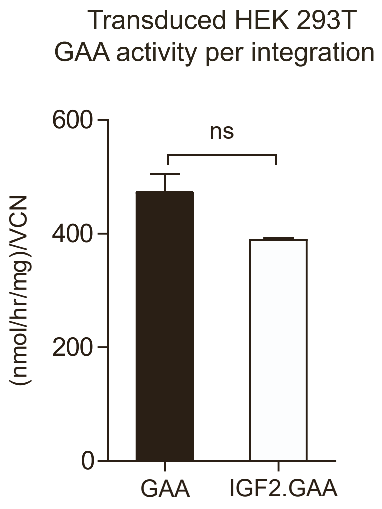

Figure S1. GAA enzyme activity per vector copy number in LV-*GAAco* or LV-*IGF2.GAAco* transduced HEK 293T. GAA activity per integration copy in LV-*GAAco* or LV-*IGF2.GAAco* transduced HEK 293T cells. Data were normalized using *HIV* and *Gapdh* loci and represent means ± SEM of three biological replicates. VCN is not normalized for chimerism. Mann-Whitney U test was used for analysis. ns, not significant.

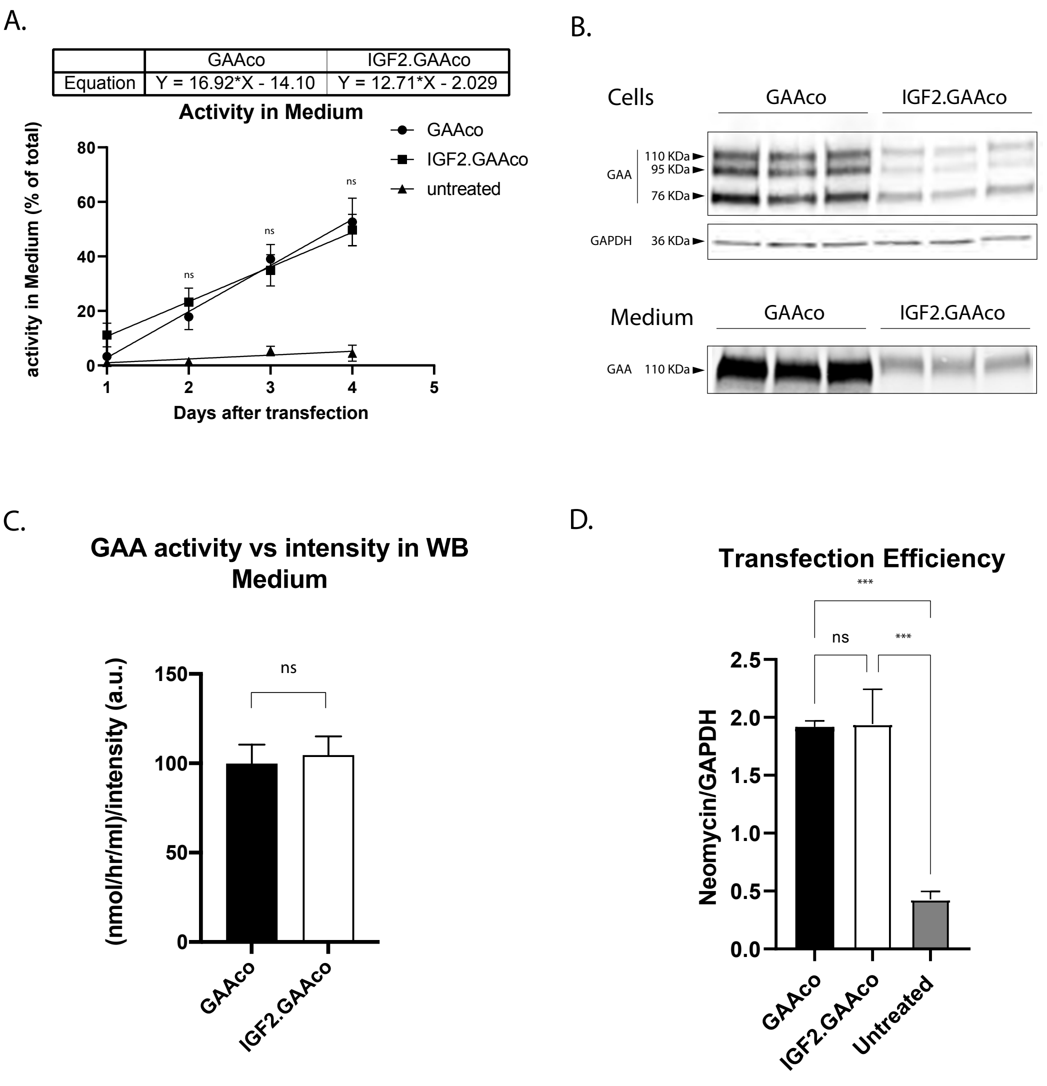

Figure S2 *In vitro* analysis of GAA and IGF2.GAA secretion.

pcDNA3.1 constructs expressing GAA or IGF2.GAA were transiently transfected in HEK 293T cells and medium and cells were analyzed. (A) Percentage of secreted GAA activity over 4 days after transient transfection. Fitting equations are shown. (B) Immunoblot analysis using an antibody to human GAA at day 4 after transfection. (C) GAA activity levels per protein levels measured by immunblot analysis in (A) in the medium at day 4 after transfection. (D) Transfection efficiency based on mRNA expression of the *Neomycin* resistance cassette present in pcDNA3.1. We note that HEK 293T cell have a Neomycin resistance and therefore displays background Neomycin expression. Data represent means ± SEM. (A and D) were analyzed by one-way ANOVA followed by Bonferroni's multiple testing correction. In (C) data were analyzed by Mann-Whitney U test. n=3 biological replicates/condition. ***$P \leq 0.001$; ns, not significant. Comparisons are indicated by brackets.

A

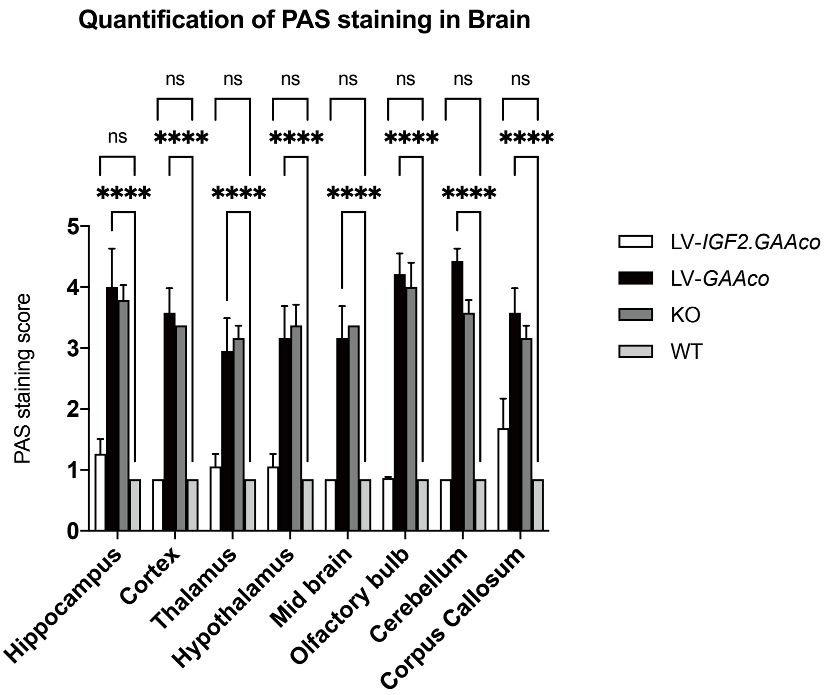

B **Rating system for pathological changes in brain after PAS staining**

Quantification is performed at 20x magnification, approximately 0.5 mm^2

PAS intensity
No staining
Staining in very few areas (less than 30 % of the field)
Staining in some areas (30 % to 60 % of the field)
Significant staining and vacuolization in many areas (more than 60 % of the field)
Strong staining and vacuolization throughout the entire field
Very strong staining and vacuolization throughout the entire field

Figure S3. Scoring of PAS reactivity in brain after gene therapy.

(A) PAS reactivity scored in hippocampus, cortex, thalamus, hypothalamus, midbrain, olfactory bulb, cerebellum and corpus callosum after high dose gene therapy (MOI 7, 10^6 Lin$^-$ transplanted cells, 9 Gy TBI). (B) Scoring system used for scoring of PAS staining in brain. Data are analyzed by two-way ANOVA with Bonferroni's correction, using vector (LV-*GAA co* or LV-*IGF2.GAAco*) and gene therapy dose as categorical variables. Results are indicat ed by brackets. *n* = 2. ns, not significant; ***P≤0 .001, ****P≤0 .0001. Scale bar = 0,1 mm

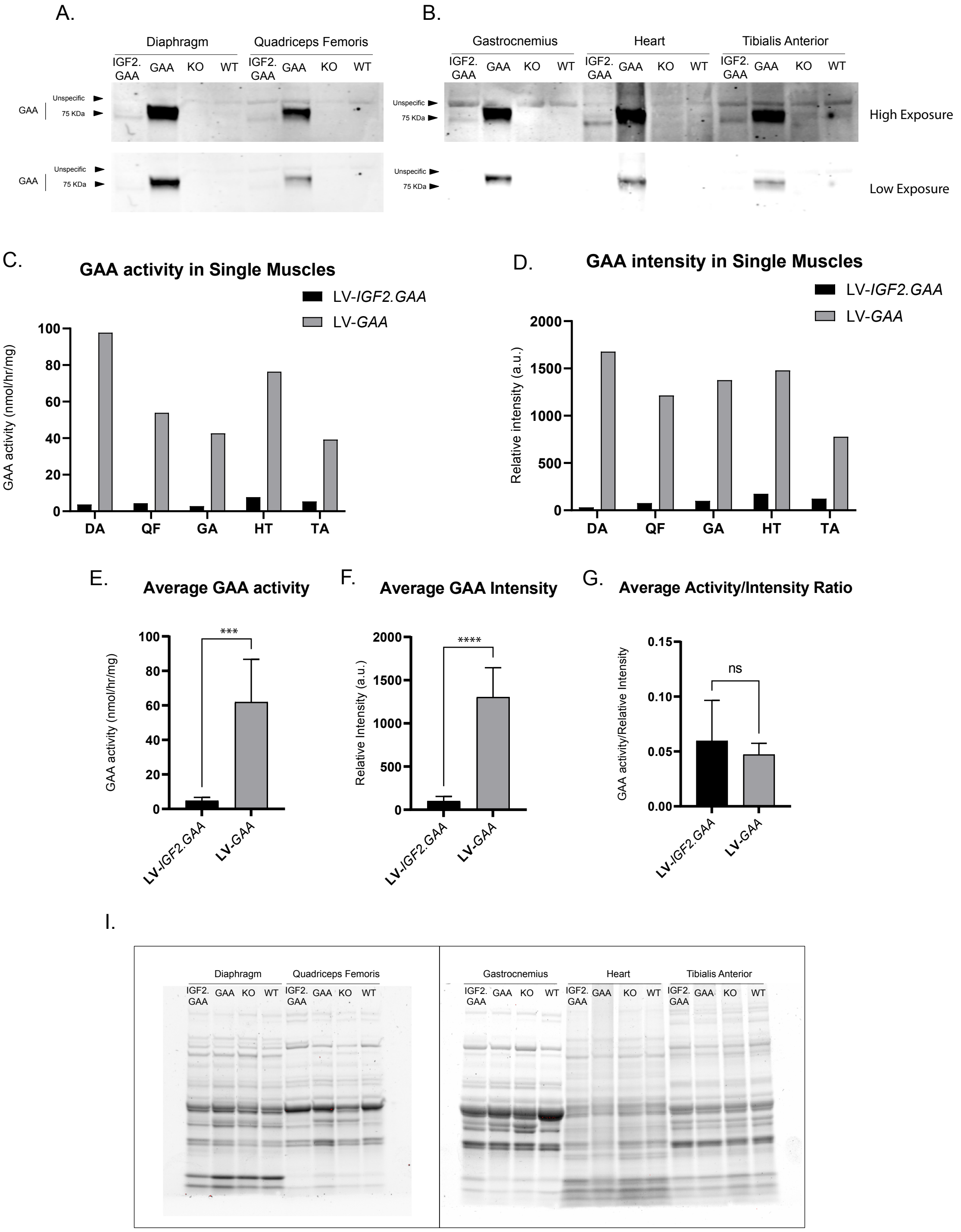
A.
Diaphragm
Quadriceps Femoris
IGF2. GAA
GAA
KO
WT
GAA
Unspecific
75 KDa
B.
Gastrocnemius
Heart
Tibialis Anterior
High Exposure
Low Exposure
C.
GAA activity in Single Muscles
LV-IGF2.GAA
LV-GAA
GAA activity (nmol/hr/mg)
DA
QF
GA
HT
TA
D.
GAA intensity in Single Muscles
Relative intensity (a.u.)
E.
Average GAA activity
***
F.
Average GAA Intensity
****
Relative Intensity (a.u.)
G.
Average Activity/Intensity Ratio
ns
GAA activity/Relative Intensity
I.

J.

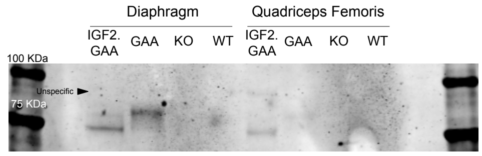

K.

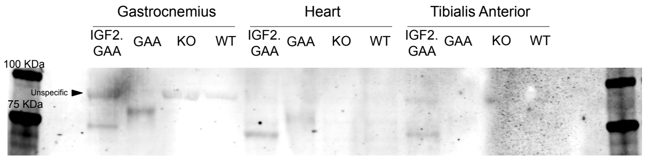

L.

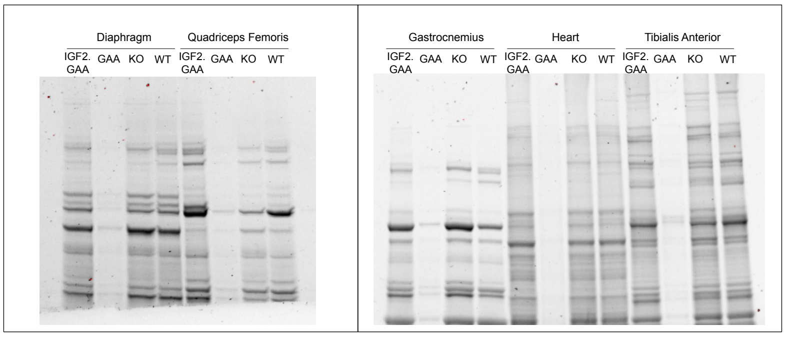

Figure S4. Immunoblot analysis of GAA protein in muscles.

(A, B) Immunoblot analysis using an antibody to human GAA in diaphragm, quadriceps femoris, gastrocnemius, heart and tibialis anterior after high dose gene therapy (MOI 7, 9 Gy, 10^6 transplanted cells). Age-matched KO and WT animals were taken as control. (C, E) GAA enzyme activity levels in the samples analyzed in (A) and (B). (D, F) Density levels of GAA were quantified from (A) and (B). Equal loading was determined by quantification of the total bands using the stain-free signal on the same gel used for the immunoblot analysis (I).

(G) GAA activity levels per protein levels measured by immunblot analysis in (A) and (B). (J, K) Immunoblot analysis as in (A) and (B) using 10-times less total protein from LV-GAA treated mice to highlight differences in the apparent molecular weight of the GAA protein. Loading levels are shown in (L) using the stain-free signal of the same gel used for the immunoblot analysis. Data information: Data represent means ± SEM and are analyzed by Mann-Whitney U-test. ****$P \leq$ 0.0001; ***$P \leq$ 0.001; ns, not significant. Comparisons are indicated by brackets.

A. Tibialis anterior

P62

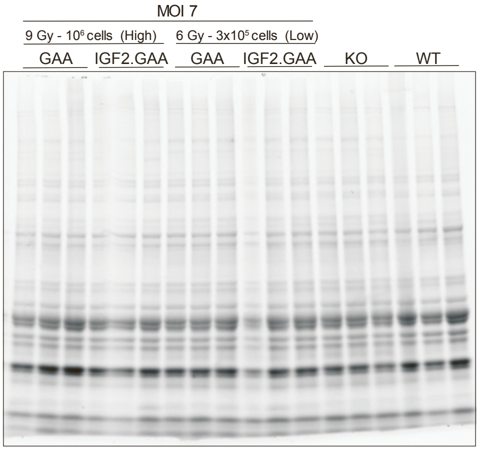

LC3 and Beclin 1

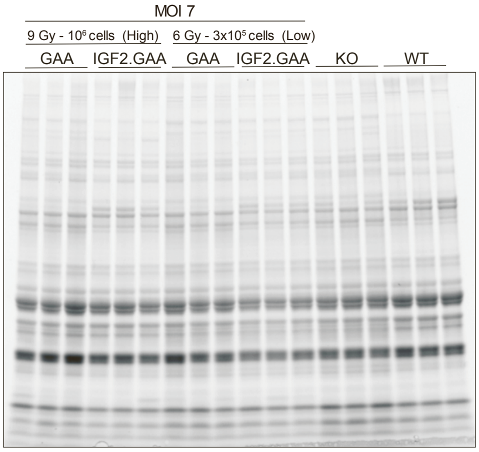

B. Heart

P62 and LC3

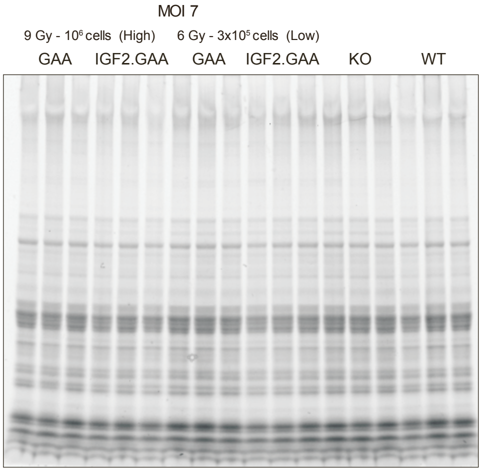

Beclin 1 (30ug)

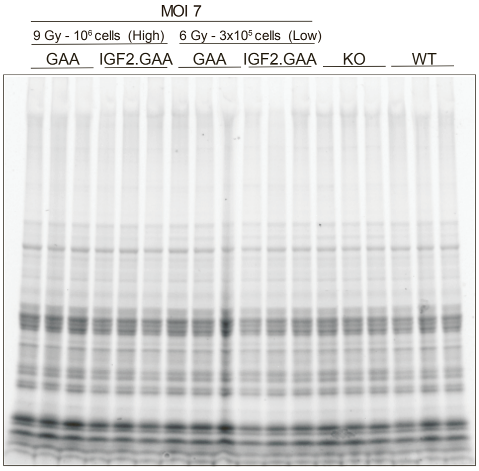

C. Brain

P62

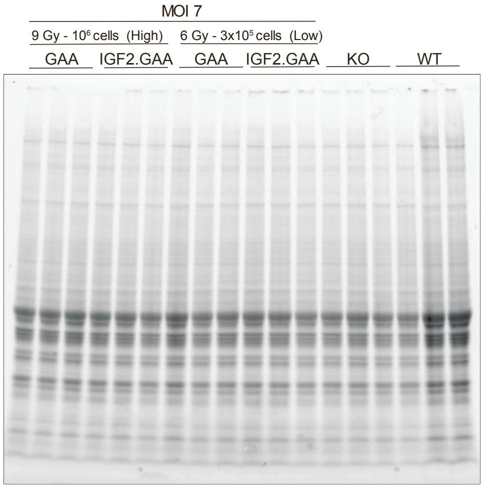

LC3 and Beclin 1

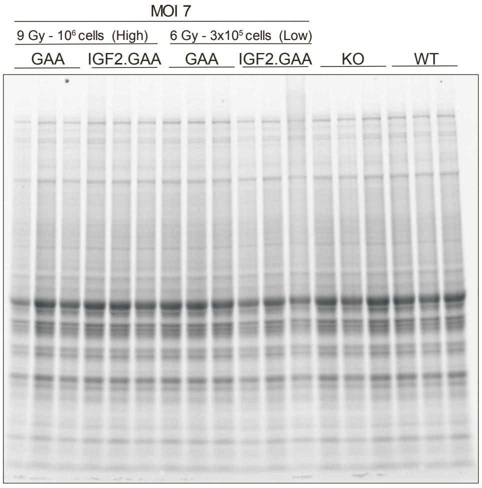

Figure S5. Total protein detection of autophagy immunoblots. Total protein load of tibialis anterior (A), heart (B) and cerebrum (C) homogenates used for autophagy immunoblots. Equal loading was determined by quantification of the total bands using the stain-free signal on the same gel used for the immunoblot analysis.

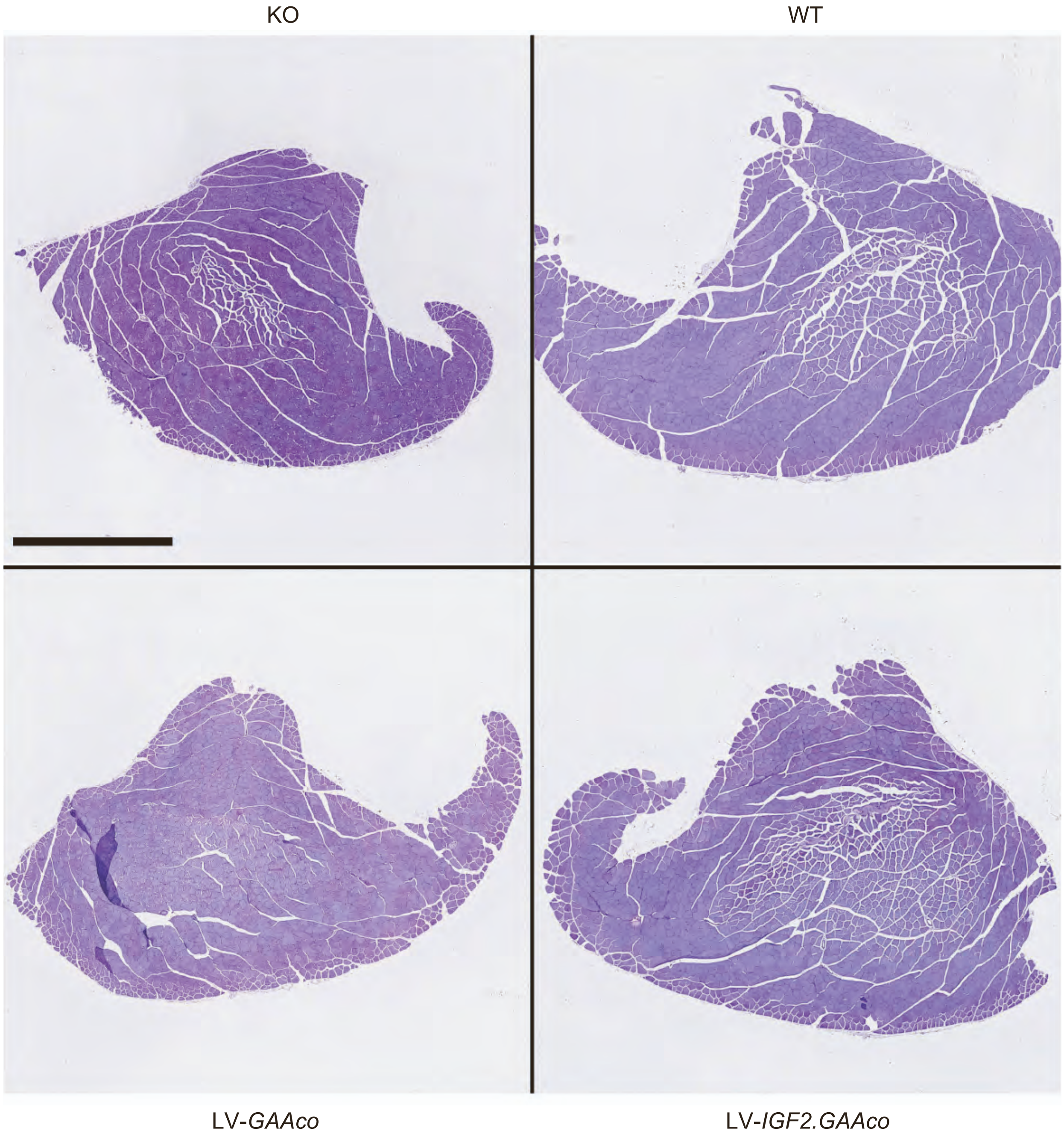

Figure S6. Glycogen reduction in tibialis anterior after gene therapy. Representative pictures of periodic acid Schiff (PAS) staining of tibialis anterior harvested from animals treated with high dose gene therapy. LV-*GAAco* and LV-*IGF2.GAAco*, *n* = 3 per group; KO, *n* = 2; WT, *n* = 2. Scale bar = 1 mm.

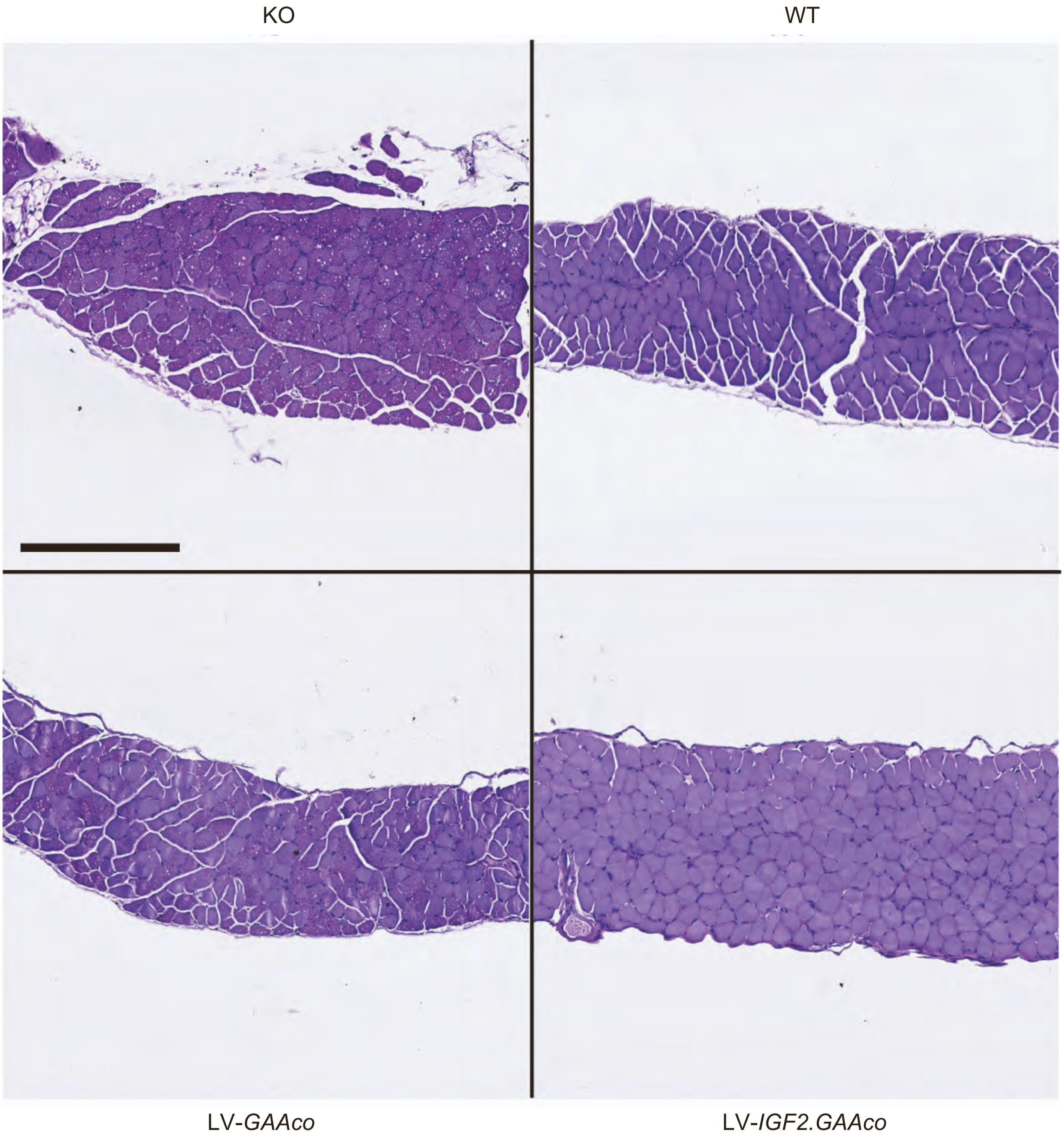

Figure S7. Glycogen reduction in diaphragm after gene therapy. Diaphragm was harvested from animals treated with high dose gene therapy and representative images of periodic acid Schiff (PAS) staining are shown. LV-*GAAco* and LV-*IGF2.GAAco* $n = 3$ per group; KO, $n = 2$; WT, $n = 2$. Scale bar = 0.25 mm.

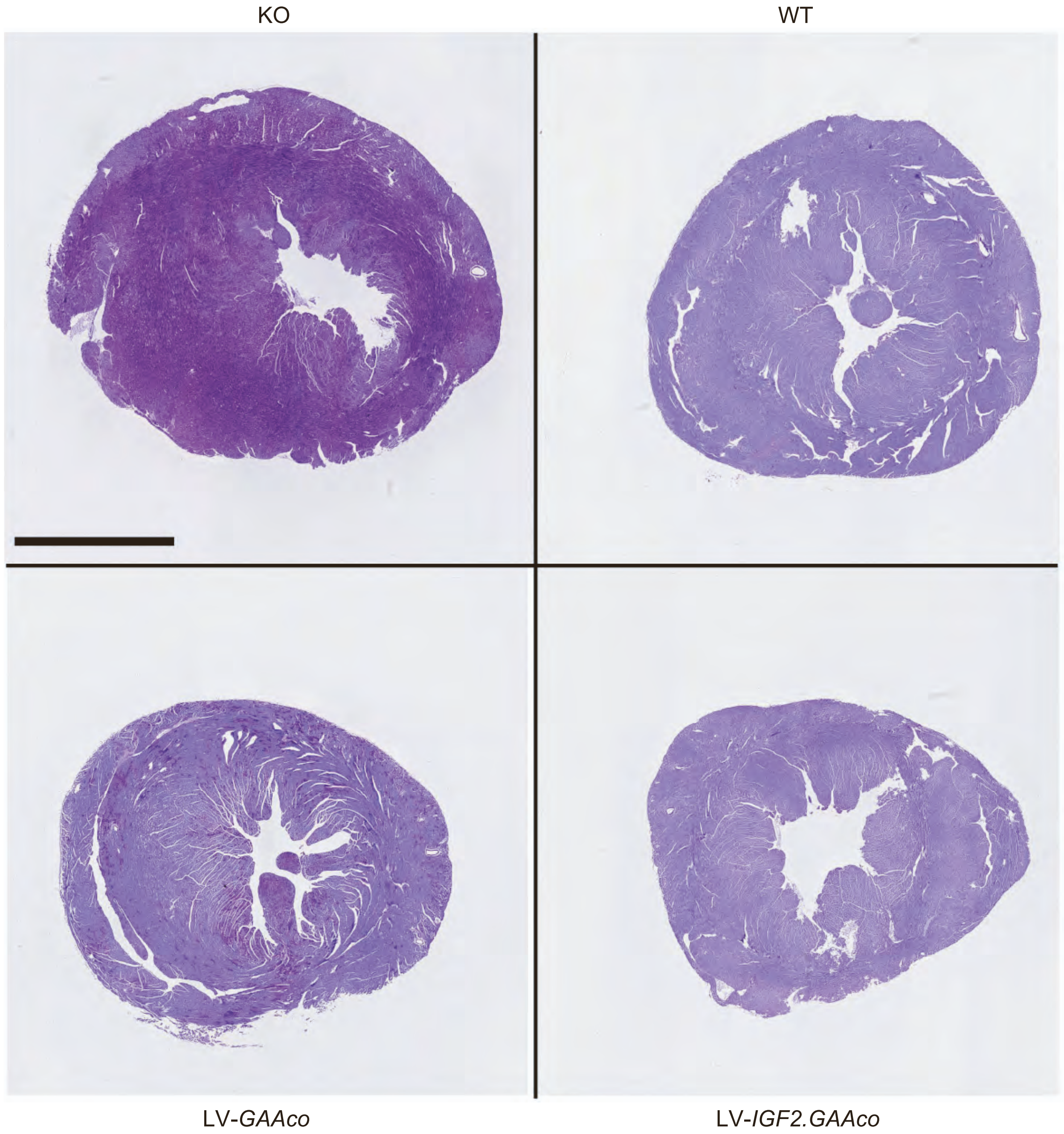

Figure S8. Glycogen reduction in cardiac tissue after gene therapy. Representative pictures of periodic acid Schiff (PAS) staining of heart harvested from animals treated with high dose gene therapy. LV-*GAAco* and LV-*IGF2.GAAco* $n = 3$ per group; KO, $n = 2$; WT, $n = 2$. Scale bar = 2 mm.

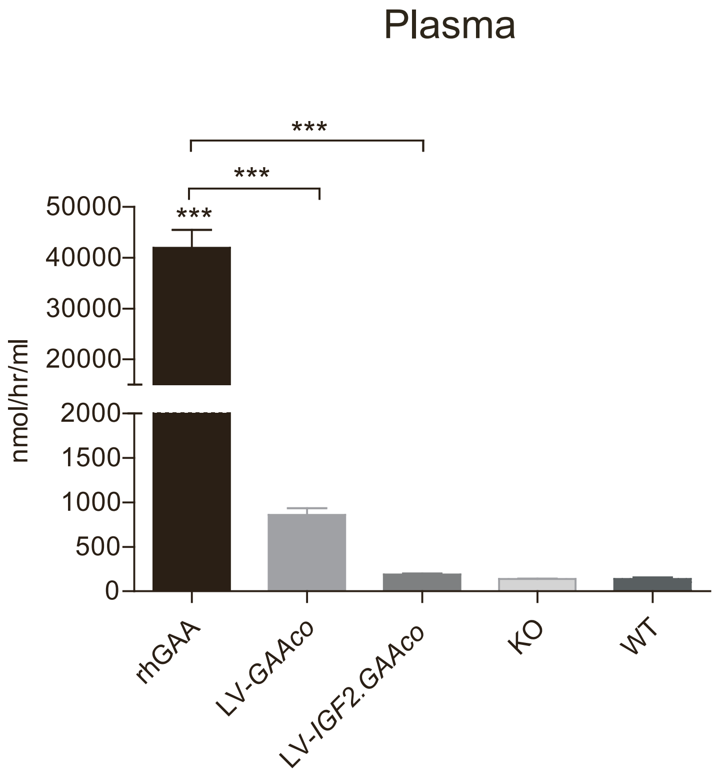

Figure S9. Enzyme activity in plasma. GAA activity in plasma was determined monthly in high dose gene therapy-treated mice and ERT-treated mice (intravenously injected with 20 mg/kg rhGAA (Myozyme); plasma was harvested 5 min after injection). Results are presented as means ± SEM. n = 3 per group; One-way ANOVA with Bonferroni's multiple correction was performed, and statistical comparisons to KO and to ERT (brackets) are shown. ns, not significant; ***$P \leq 0.001$.

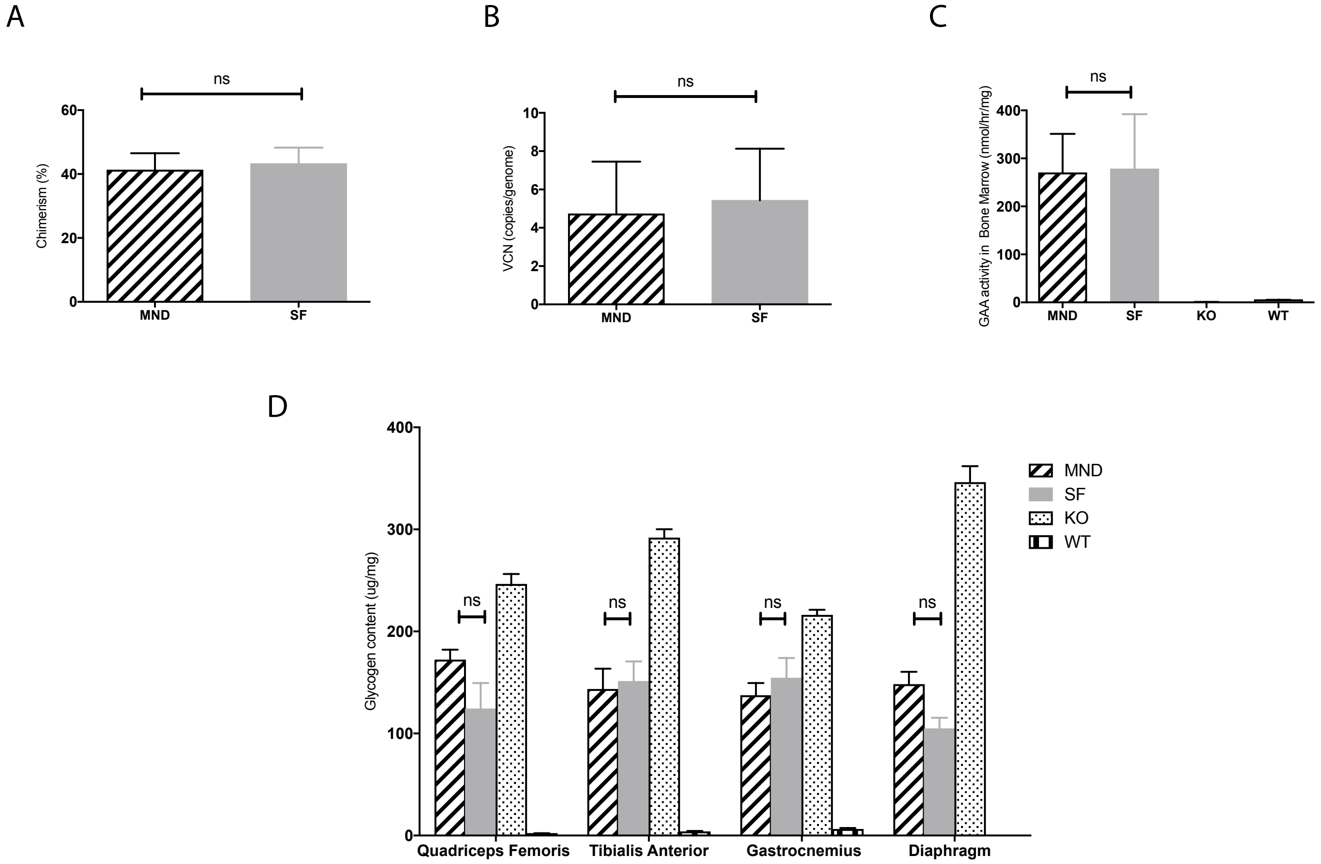

Figure S10. The SF promoter or the MND promoter drive a similar therapeutic outcome in muscles after low dose gene therapy with LV-*GAAco*.

(A) Chimerism, expressed as the percentage of reconstituted male donor cells in bone marrow of female recipients treated with gene therapy, determined by qPCR on *Sry* and *Gapdh* loci. (B) VCN measured in bone marrow by qPCR on *HIV* and *Gapdh* loci. VCN is not normalized for chimerism. (C) GAA activity in bone marrow after low dose gene therapy (MOI 7, 10^6 Lin$^-$ transplanted cells, 6 Gy TBI) with LV-*GAAco* with MND or SF promoters. (D) Total glycogen content in skeletal muscles after f gene therapy with MND LV-*GAAco* or SF LV-*GAAco*. Data are presented as means ± SEM. In (A, B, C) data are analyzed by Mann-Whitney U test. Comparison between SF and MND promoters is indicated. In (D) data are analyzed by two-way ANOVA followed by Bonferroni's multiple testing correction, using promoter (MND or SF) and skeletal muscle analysed as categorical variables. Significance is expressed as relative to WT; other significant comparisons are indicated by brackets. *n* = 5. ns, not significant.

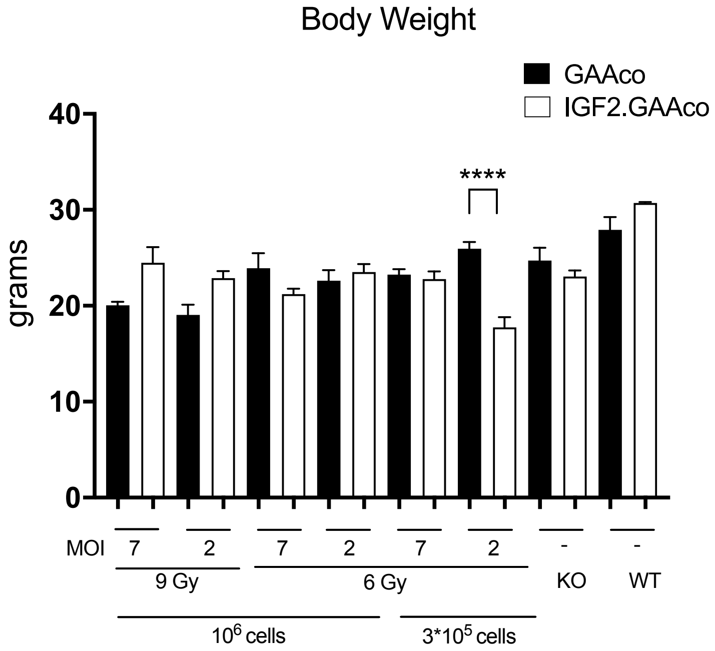

Figure S11. Body weight after gene therapy. Body weight after gene therapy with LV-*GAAco* and LV-*IGF2.GAAco*. LV-*GAAco* and LV-*IGF2.GAAco*, $n = 7$ per group; KO, $n = 5$; WT, $n = 5$. ns, not significant; ****$P \leq 0.0001$; two-way ANOVA with Bonferroni's correction. Data are presented as means ± SEM.

Table S1. Sequence of primers for qPCR

Primers	Sequence
HIV-U3 forward	5′-CTGGAAGGGCTAATTCACTC-3′
HIV-PSI reverse	5′-GGTTTCCCTTTCGCTTTCAG-3′
Sry forward	5′-TCATCGGAGGGCTAAAGTGTCAC-3′
Sry reverse	5′-TGGCATGTGGGTTCCTGTCC-3′
Gapdh forward (mouse)	5′-TAATGGGGAGAGGTTCGATG-3′
Gapdh reverse (mouse)	5′-GCTGCTTCCCGAGTAAAATG-3′
GAPDH forward (human)	5′-CGGTTTCTATAAATTGAGCCCG-3′
GAPDH reverse (human)	5′-GCGACGCAAAAGAAGATGC-3′

Table S2. Nonlinear regression model for the relationship between vector copy number and glycogen content for LV-IGF2.GAAco and LV-GAAco treted mice.

Tissues	Estimates	Std. Error	95% Confidence Interval		Statistic outcome
			Lower Bound	Upper Bound	
Heart	-0.556	0.183	-0.919	-0.193	Significant
Tibialis anterior	-0.408	0.131	-0.668	-0.148	Significant
Cerebrum	-0.490	0.165	-0.817	-0.163	Significant

The difference in the exponential decay rate (λ) between LV-*IGF2co.GAAco* and LV-*GAAco* is defined significant when the 95% confidence interval for B1 does not contain zero

Table S3. Statistical analysis and group comparison for glycogen assays, VCN and chimerism in bone marrow.

Data are analysed by two-way ANOVA with Bonferroni's correction using vector type - LV-GAAco or LV-IGF2co.GAAco - and gene therapy dose - combination of irradiation dose, amount of transplanted cells and MOI - as categorical variables.

Comparison	Glycogen - Tibialis Antirior		Glycogen - Quadriceps Femoris		Glycogen - Diaphragm		Glycogen - Gastrocnemius		Glycogen - Heart		Glycogen - Cerebellum		Glycogen - Cerebrum		Vector Copy Number		Chimerism	
Do Vector type or Gene therapy dose influece the quantitative variable under study?	Summary	Adjusted P Value	Summary	Adjusted P Value	Summary	Adjusted P Value	Summary	Adjusted P Value	Summary	Adjusted P Value	Summary	Adjusted P Value	Summary	Adjusted P Value	Summary	Adjusted P Value	Summary	Adjusted P Value
Gene Therapy Dose	****	<0,0001	****	<0,0001	****	<0,0001	****	<0,0001	****	<0,0001	****	<0,0001	****	<0,0001	****	<0,0001	****	>0,0001
Vector (LV-GAAco or LV-IGF2.GAAco)	****	<0,0001	****	<0,0001	****	<0,0001	****	<0,0001	****	<0,0001	****	<0,0001	****	<0,0001	****	<0,0001	**	0,0017
LV-GAAco vs LV-IGF2.GAAco	Summary	Adjusted P Value	Summary	Adjusted P Value	Summary	Adjusted P Value	Summary	Adjusted P Value	Summary	Adjusted P Value	Summary	Adjusted P Value	Summary	Adjusted P Value	Summary	Adjusted P Value	Summary	Adjusted P Value
9Gy-10^6 cells-MOI 7	****	<0,0001	****	<0,0001	****	<0,0001	****	<0,0001	****	<0,0001	****	<0,0001	****	<0,0001	****	<0,0001	ns	>0,9999
9Gy-10^6 cells-MOI 2	***	0,0007	****	<0,0001	****	<0,0001	****	<0,0001	****	<0,0001	****	<0,0001	****	<0,0001	ns	>0,9999	ns	>0,9999
6Gy-10^6 cells-MOI 7	ns	>0,9999	****	<0,0001	**	0,0025	*	0,0209	ns	>0,9999	****	<0,0001	****	<0,0001	*	0,0142	ns	0,0608
6Gy-10^6 cells-MOI 2	ns	0,8656	****	<0,0001	****	<0,0001	**	0,0013	**	0,0098	****	<0,0001	****	<0,0001	*	0,0212	*	0,0352
6Gy-10^6 cells-MOI 7	ns	>0,9999	ns	>0,9999	ns	>0,9999	ns	>0,9999	ns	>0,9999	ns	0,1927	***	0,0003	ns	0,6029	ns	>0,9999
6Gy-10^6 cells-MOI 2	ns	>0,9999	ns	>0,9999	ns	>0,9999	ns	>0,9999	ns	>0,9999	*	0,0129	****	<0,0001	ns	0,4697	ns	0,0639
KO	ns	>0,9999	ns	>0,9999	ns	>0,9999	ns	0,8452	ns	>0,9999	ns	>0,9999	ns	>0,9999				
WT	*	0,0313	ns	>0,9999	ns	0,4067	ns	>0,9999	ns	>0,9999	****	<0,0001	ns	>0,9999				
Treatment vs WT	Summary	Adjusted P Value	Summary	Adjusted P Value	Summary	Adjusted P Value	Summary	Adjusted P Value	Summary	Adjusted P Value	Summary	Adjusted P Value	Summary	Adjusted P Value	Summary	Adjusted P Value	Summary	Adjusted P Value
GAAco 9Gy-10^6 cells-MOI 7 vs WT	*	0,0118	****	<0,0001	****	<0,0001	****	<0,0001	****	<0,0001	****	<0,0001	****	<0,0001				
IGF2.GAAco 9Gy-10^6 cells-MOI 7 vs WT	ns	>0,9999	**	0,0011	ns	>0,9999	ns	>0,9999	ns	>0,9999	ns	>0,9999	ns	>0,9999				
GAAco 9Gy-10^6 cells- MOI 2 vs WT	****	<0,0001	****	<0,0001	****	<0,0001	****	<0,0001	****	<0,0001	****	<0,0001	****	<0,0001				
IGF2.GAAco 9Gy-10^6 cells-MOI 2 vs WT	***	0,0001	****	<0,0001	****	<0,0001	****	<0,0001	ns	>0,9999	ns	>0,9999	ns	>0,9999				
GAAco 6Gy-10^6 cells-MOI 7 vs WT	****	<0,0001	****	<0,0001	****	<0,0001	****	<0,0001	****	<0,0001	****	<0,0001	****	<0,0001				
IGF2.GAAco 6Gy-10^6 cells-MOI 7 vs WT	****	<0,0001	****	<0,0001	****	<0,0001	****	<0,0001	****	<0,0001	***	0,0003	****	<0,0001				
GAAco 6Gy-10^6 cells-MOI 2 vs WT	****	<0,0001	****	<0,0001	****	<0,0001	****	<0,0001	****	<0,0001	****	<0,0001	****	<0,0001				
IGF2.GAAco 6Gy-10^6 cells-MOI 2 vs WT	****	<0,0001	****	<0,0001	****	<0,0001	****	<0,0001	****	<0,0001	***	0,0002	****	<0,0001				
GAAco 6Gy-3*10^5 cells-MOI 7 vs WT	****	<0,0001	****	<0,0001	****	<0,0001	****	<0,0001	****	<0,0001	****	<0,0001	****	<0,0001				
IGF2.GAAco 6Gy-3*10^5 cells-MOI 7 vs WT	****	<0,0001	****	<0,0001	****	<0,0001	****	<0,0001	****	<0,0001	****	<0,0001	****	<0,0001				
GAAco 6Gy-3*10^5 cells-MOI 2 vs WT	****	<0,0001	****	<0,0001	****	<0,0001	****	<0,0001	****	<0,0001	****	<0,0001	****	<0,0001				
IGF2.GAAco 6Gy-3*10^5 cells-MOI 2 vs WT	****	<0,0001	****	<0,0001	****	<0,0001	****	<0,0001	****	<0,0001	****	<0,0001	****	<0,0001				
GAAco KO vs WT	****	<0,0001	****	<0,0001	****	<0,0001	****	<0,0001	****	<0,0001	****	<0,0001	****	<0,0001				
IGF2.GAAco KO vs WT	****	<0,0001	****	<0,0001	****	<0,0001	****	<0,0001	****	<0,0001	****	<0,0001	****	<0,0001				
Treatment vs KO	Summary	Adjusted P Value	Summary	Adjusted P Value	Summary	Adjusted P Value	Summary	Adjusted P Value	Summary	Adjusted P Value	Summary	Adjusted P Value	Summary	Adjusted P Value	Summary	Adjusted P Value	Summary	Adjusted P Value
GAAco 9Gy-10^6 cells-MOI 7 vs KO	****	<0,0001	****	<0,0001	****	<0,0001	****	<0,0001	****	<0,0001	ns	>0,9999	ns	>0,9999				
IGF2.GAAco 9Gy-10^6 cells-MOI 7 vs KO	****	<0,0001	****	<0,0001	****	<0,0001	****	<0,0001	****	<0,0001	****	<0,0001	****	<0,0001				
GAAco 9Gy-10^6 cells- MOI 2 vs KO	ns	0,258	****	<0,0001	**	0,0083	****	<0,0001	**	0,0035	ns	>0,9999	ns	>0,9999				
IGF2.GAAco 9Gy-10^6 cells-MOI 2 vs KO	****	<0,0001	****	<0,0001	****	<0,0001	****	<0,0001	****	<0,0001	****	<0,0001	****	<0,0001				
GAAco 6Gy-10^6 cells-MOI 7 vs KO	ns	0,2014	****	<0,0001	****	<0,0001	****	<0,0001	****	<0,0001	ns	>0,9999	ns	>0,9999				
IGF2.GAAco 6Gy-10^6 cells-MOI 7 vs KO	*	0,0276	****	<0,0001	****	<0,0001	****	<0,0001	****	<0,0001	****	<0,0001	****	<0,0001				
GAAco 6Gy-10^6 cells-MOI 2 vs KO	ns	0,4284	****	<0,0001	**	0,0013	****	<0,0001	**	0,0034	ns	>0,9999	ns	>0,9999				
IGF2.GAAco 6Gy-10^6 cells-MOI 2 vs KO	**	0,0041	****	<0,0001	****	<0,0001	****	<0,0001	****	<0,0001	****	<0,0001	****	<0,0001				
GAAco 6Gy-3*10^5 cells-MOI 7 vs KO	ns	>0,9999	ns	0,1072	ns	0,2074	**	0,0075	*	0,0481	ns	>0,9999	ns	>0,9999				
IGF2.GAAco 6Gy-3*10^5 cells-MOI 7 vs KO	ns	>0,9999	ns	0,1912	ns	0,2227	ns	0,0818	ns	0,0733	ns	0,24	***	0,0003				
GAAco 6Gy-3*10^5 cells-MOI 2 vs KO	ns	>0,9999	***	0,0005	ns	0,0673	****	<0,0001	**	0,007	ns	>0,9999	ns	>0,9999				
IGF2.GAAco 6Gy-3*10^5 cells-MOI 2 vs KO	ns	>0,9999	***	0,0004	*	0,0102	***	0,0005	***	0,0007	**	0,0077	****	<0,0001				
9 Gy vs 6 Gy	Summary	Adjusted P Value	Summary	Adjusted P Value	Summary	Adjusted P Value	Summary	Adjusted P Value	Summary	Adjusted P Value	Summary	Adjusted P Value	Summary	Adjusted P Value	Summary	Adjusted P Value	Summary	Adjusted P Value
GAAco-9Gy-10^6 cells-MOI 7 vs 6Gy-10^6 cells-MOI 7	ns	0,1246	****	<0,0001	*	0,0265	*	0,0389	ns	>0,9999	ns	>0,9999	ns	>0,9999	****	<0,0001	****	<0,0001
GAAco-9Gy-10^6 cells-MOI 2 vs 6Gy-10^6 cells-MOI 2	ns	>0,9999	ns	>0,9999	ns	>0,9999	ns	>0,9999	ns	>0,9999	ns	>0,9999	ns	>0,9999	ns	>0,9999	**	0,0017
IGF2.GAAco-9Gy-10^6 cells-MOI 7 vs 6Gy-10^6 cells-MOI 7	****	<0,0001	****	<0,0001	****	<0,0001	****	<0,0001	****	<0,0001	****	<0,0001	****	<0,0001	****	<0,0001	****	<0,0001
IGF2.GAAco-9Gy-10^6 cells-MOI 2 vs 6Gy-10^6 cells- MOI 2	**	0,0037	****	<0,0001	****	<0,0001	****	<0,0001	****	<0,0001	****	<0,0001	****	<0,0001	ns	0,1305	****	<0,0001
MOI 7 vs MOI 2	Summary	Adjusted P Value	Summary	Adjusted P Value	Summary	Adjusted P Value	Summary	Adjusted P Value	Summary	Adjusted P Value	Summary	Adjusted P Value	Summary	Adjusted P Value	Summary	Adjusted P Value	Summary	Adjusted P Value
GAAco-9Gy-10^6 cells-MOI 7 vs 9Gy-10^6 cells-MOI 2	ns	>0,9999	**	0,0098	*	0,0285	ns	>0,9999	ns	0,5735	ns	>0,9999	ns	>0,9999	****	<0,0001	ns	>0,9999
GAAco-6Gy-10^6 cells-MOI 7 vs 6Gy-10^6 cells-MOI 2	ns	>0,9999	ns	>0,9999	ns	>0,9999	ns	>0,9999	ns	>0,9999	ns	>0,9999	ns	>0,9999	ns	>0,9999	*	0,0333
GAAco-6Gy-3*10^5 cells-MOI 7 vs 6Gy-3*10^5 cells-MOI 2	ns	>0,9999	ns	>0,9999	ns	>0,9999	ns	>0,9999	ns	>0,9999	ns	>0,9999	ns	>0,9999	ns	>0,9999	*	0,0404
IGF2.GAAco-9Gy-10^6 cells-MOI 7 vs 9Gy-10^6 cells-MOI 2	*	0,033	ns	0,2958	****	<0,0001	***	0,0001	ns	0,9553	ns	>0,9999	ns	0,3697	ns	0,5609	ns	>0,9999
IGF2.GAAco-6Gy-10^6 cells-MOI 7 vs 6Gy-10^6 cells-MOI 2	ns	>0,9999	ns	>0,9999	ns	>0,9999	ns	>0,9999	ns	>0,9999	ns	>0,9999	ns	0,9462	ns	>0,9999	ns	0,06
IGF2.GAAco-6Gy-3*10^5 cells-MOI 7 vs 6Gy-3*10^5 cells-MOI 2	ns	>0,9999	ns	>0,9999	ns	>0,9999	ns	>0,9999	ns	>0,9999	ns	>0,9999	ns	>0,9999	ns	>0,9999	ns	>0,9999
10^6 cells vs 3*10^5 cells	Summary	Adjusted P Value	Summary	Adjusted P Value	Summary	Adjusted P Value	Summary	Adjusted P Value	Summary	Adjusted P Value	Summary	Adjusted P Value	Summary	Adjusted P Value	Summary	Adjusted P Value	Summary	Adjusted P Value
GAAco-6Gy-10^6 cells-MOI 7 vs 6Gy-3*10^5 cells-MOI 7	ns	>0,9999	ns	0,1122	ns	0,5419	*	0,0251	ns	0,2807	ns	>0,9999	ns	>0,9999	ns	0,8009	ns	0,0804
GAAco-6Gy-10^6 cells-MOI 2 vs 6Gy-3*10^5 cells-MOI 2	ns	>0,9999	ns	0,3615	ns	>0,9999	ns	0,1238	ns	>0,9999	ns	>0,9999	ns	>0,9999	ns	0,3938	****	<0,0001
IGF2.GAAco-6Gy-10^6 cells-MOI 7 vs 6Gy-3*10^5 cells-MOI 7	ns	0,4885	****	<0,0001	****	<0,0001	****	<0,0001	ns	0,0606	**	0,0086	****	<0,0001	ns	>0,9999	ns	>0,9999
IGF2.GAAco-6Gy-10^6 cells-MOI 2 vs 6Gy-3*10^5 cells-MOI 2	ns	>0,9999	****	<0,0001	****	<0,0001	***	0,0004	ns	0,1211	ns	0,0745	***	0,0002	ns	>0,9999	ns	0,1307
